# Overview on Molecular Biomarkers for Laryngeal Cancer: Looking for New Answers to an Old Problem

**DOI:** 10.3390/cancers14071716

**Published:** 2022-03-28

**Authors:** Michela Falco, Chiara Tammaro, Takashi Takeuchi, Alessia Maria Cossu, Giuseppe Scafuro, Silvia Zappavigna, Annalisa Itro, Raffaele Addeo, Marianna Scrima, Angela Lombardi, Filippo Ricciardiello, Carlo Irace, Michele Caraglia, Gabriella Misso

**Affiliations:** 1 Department of Precision Medicine, University of Campania “Luigi Vanvitelli”, 80138 Naples, Italy; michela.falco@unicampania.it (M.F.); chiara.tammaro@unicampania.it (C.T.); takashi19900706@gmail.com (T.T.); alessiamaria.cossu@biogem.it (A.M.C.); giuseppe.scafuro@unicampania.it (G.S.); silvia.zappavigna@unicampania.it (S.Z.); angelalombardi@hotmail.it (A.L.); michele.caraglia@unicampania.it (M.C.); 2 Molecular Diagnostics Division, Wakunaga Pharmaceutical Co., Ltd., Hiroshima 739-1195, Japan; 3 Laboratory of Molecular and Precision Oncology, Biogem Scarl, Institute of Genetic Research, 83031 Ariano Irpino, Italy; marianna.scrima@biogem.it; 4 Department of Advanced Medical and Surgical Sciences, University of Campania “Luigi Vanvitelli”, 80138 Naples, Italy; annalisa.itro@unicampania.it; 5 Oncology Operative Unit, Hospital of Frattamaggiore, ASLNA-2NORD, 80020 Naples, Italy; raffaeleaddeo19@gmail.com; 6 Division of Otorhinolaryngology, “A. Cardarelli” Hospital, 80131 Naples, Italy; filippo.ricciardiello@aocardarelli.it; 7 Department of Pharmacy, School of Medicine and Surgery, University of Naples “Federico II”, 80131 Naples, Italy; carlo.irace@unina.it

**Keywords:** LSCC (laryngeal squamous cell carcinoma), miRNA, lncRNA, biomarkers, epigenetic modifications, ctDNA, CTC, inflammatory markers, TME (tumor microenvironment)

## Abstract

**Simple Summary:**

Laryngeal cancer represents one of the most common head and neck cancers worldwide. It can arise in different larynx subsites, each associated with specific clinical-pathological characteristics and treatment options. Due to the nonspecific symptoms, laryngeal cancer diagnosis often occurs in a late phase, resulting in delayed treatment and worse prognosis. Therefore, successful clinical management is strictly linked to the identification of reliable diagnostic and prognostic biomarkers. Herein, we provide an overview of the most promising molecular factors to date identified for both detection and monitoring, focusing on mutated genes, non-coding RNAs, transforming epigenetic events, inflammatory mediators, and immune-related agents.

**Abstract:**

Laryngeal squamous cell cancer (LSCC) accounts for almost 25–30% of all head and neck squamous cell cancers and is clustered according to the affected districts, as this determines distinct tendency to recur and metastasize. A major role for numerous genetic alterations in driving the onset and progression of this neoplasm is emerging. However, major efforts are still required for the identification of molecular markers useful for both early diagnosis and prognostic definition of LSCC that is still characterized by significant morbidity and mortality. Non-coding RNAs appear the most promising as they circulate in all the biological fluids allowing liquid biopsy determination, as well as due to their quick and characteristic modulation useful for non-invasive detection and monitoring of cancer. Other critical aspects are related to recent progress in circulating tumor cells and DNA detection, in metastatic status and chemo-refractoriness prediction, and in the functional interaction of LSCC with chronic inflammation and innate immunity. We review all these aspects taking into account the progress of the technologies in the field of next generation sequencing.

## 1. Introduction

Laryngeal cancer represents one-third of all head and neck cancers which are the sixth most common cancer worldwide, occurring in different subsites of larynx, with different symptoms and treatments [1]. In 2020, 184,615 new cases were diagnosed globally, while 99,840 deaths were recorded [2]. Based on the data coming from the Surveillance, Epidemiology, and End Results (SEER) Program, from 2011 to 2017, the estimated 5-year relative survival for patients diagnosed for laryngeal cancer was 60.7% as also found in the last decades. One of the main factors affecting this index is represented by the high percentage of diagnosis at advanced stage. In fact, the 5-year relative survival rate for localized laryngeal cancer increases up to 77.9%. Moreover, SEER data report a rate of new cases of 4.9 per 100,000 for males and 1.1 for females, whereas the median age at diagnosis is 66 years for both sexes [3]. Alcohol, tobacco consumption, and infections are the main causative factors for LSCC. Human papilloma virus (HPV) is considered an independent risk factor and HPV16 represents the most common type in laryngeal tumors [4]. At diagnosis time, head and neck squamous cell carcinomas (HNSSCs) patients often present malnutrition and cachexia which are severe wasting conditions affecting quality of life. Both cachexia and malnutrition are correlated with poor survival or longest post-operative period [5,6]. The clinical diagnosis is firstly made by neck palpation, echography, suspension laryngoscopy, computed tomography (CT), or even magnetic resonance imaging (MRI), supplemented by a positron emission tomography (PET) in an advanced or suspected locoregional disease. All these findings contribute to assessing the clinical TMN (cTMN) classification. In addition, a detailed histopathological analysis performed on the tumor specimens defines the pathological TMN (pTMN) [7].

Early diagnosis ensures the choice from a wide range of potential successful therapeutic strategies. During the last years, many changes have occurred in LSCC treatment and a significant improvement of patients’ quality of life has been achieved. Before the 1980s, a total laryngectomy followed by radio therapy (RT) was the standard procedure for advanced tumors. Today, a dedicated multidisciplinary team (MDT) based on an accurate preoperative assessment guarantees the best treatment option. T1–T2 LSCC patients with a tumor in situ undergo organ and function preservation treatment by primary larynx-preserving surgery, transoral CO_2_ laser excision, or RT [8]. Treatment of locally advanced disease (T3–T4) involves a non-mutilating surgery strategy in combination with chemotherapy (CT). The most commonly administered drugs in LSCC CT treatment are cisplatin and 5-fluorouracil and, most recently, taxanes and monoclonal antibodies, such as cetuximab [9]. An old and invasive surgery approach, represented by total laryngectomy, is now reserved only to those patients with extensive T3–T4 stages. Furthermore, neck dissection can also occur for patients positive with lymph nodal metastases (LNM) [10].

Given the huge limitation represented by delayed diagnosis, mostly due to the absence of premonitory symptoms, there is an urgent need to find new early diagnostic and prognostic biomarkers. Similarly, the deepening of knowledge concerning the molecular mechanisms underlying the pathology could facilitate a prompt diagnosis improving LSCC patients’ quality of life.

## 2. Histopathological Features of LSCC

Currently, histopathological analysis is essential for clinicians to determine the surgeon margins. These can be hypothetically defined “safe” if negative for the presence of cancer cells. The current strategy to have an “adequate” resection margin is 5 mm from the invasive front of the carcinoma [11]. One of the most interesting recent findings underlining the importance of surgical margins is that patients with negative margins but positive for prognostic biomarkers, such as p53, Cyclin-1, Bcl-2 and others, show enhanced tendency to relapse [12].

A large part (95%) of laryngeal malignancies is represented by squamous cell carcinomas (LSCCs), while the remaining 5% are verrucous squamous cell carcinomas, chondrosarcomas, leiomyosarcomas, and others [13,14]. In recent years, there has been an increasing interest in understanding the role played by HPV in LSCC etiopathology. A great deal of studies report HPV-16 as the most common viral type detected and internalized in LSCC. Its capability to encode oncoproteins able to drive cell cycle progression has been also characterized [15]. HPV-16 positivity was found to be independently correlated with LSCC onset and, surprisingly, LSCC risk in “never-smokers” was found to be higher than in “ever-smokers” [16]. Moreover, most recently, Lifsics et al. confirmed, by means of immunohistochemistry, the involvement of p16 and E6/E7 oncoproteins in LSCC tumorigenesis, while loss of E6/E7 oncoproteins in some LSCC analyzed tissues suggested the occurrence of other tumorigenic mechanisms to be further deepened [17].

According to the American Joint Committee on Cancer (AJCC) 8th ed. (2017), the primary larynx cancer can be located at the glottic, supraglottic, or subglottic region, with incidence following the reported order [18].

The supraglottic tumors often appear silent and more invasive compared to the glottic ones which, conversely, present more chances to remain localized, mainly due to their more marked ability to establish an extensive lymphatic network. Invasiveness is a specific feature of squamous cell cancers (SCCs) which frequently spread into the stroma disrupting the basal membrane [19].

LSCC diagnosis must be necessarily preceded by an in-depth differential study, to exclude other similar and less dangerous diseases. The classical method which usually allows LSCC diagnosis is the histopathologic exam by hematoxylin and eosin staining (H&E). In the case of unclear histology, immunohistochemical methods are performed to target specific markers, such as p53, CD44, and EGFR [20]. Nevertheless, in most cases there is no need to perform immunohistochemical reactions to confirm LSCC diagnosis. The invasiveness is conventionally graded after histopathological analysis as follows: well (G1), moderately (G2), and poorly differentiated (G3). The histological confirmation of extra nodal metastatic spreading is extremely important for adjuvant treatment planning [21].

Most LSCCs are described as keratinizing due to the copious keratin production by tumor cells. An indicator of invasiveness called desmoplasia can be detected by H&E staining, which can reveal tumor cell movement from the epithelium into the stroma.

LSCC is frequently characterized by delayed diagnosis for the absence of evident signs or symptoms. However, it often develops from precancerous lesions, such as epithelial hyperplasia, epidermoid metaplasia, acanthosis, keratosis, dyskeratosis, pachydermia, and dysplasia. In particular, a strong consequential relationship has been found with chronic dysplastic conditions, with higher risk of carcinoma transformation in the case of severe dysplasia grade [22]. Multiple scientific studies assessed the histopathological steps of pathology progression. Johnson et al. showed the transition from epithelial cell hyperplasia, to dysplasia, to carcinoma in situ, and, ultimately, to invasive carcinoma [23].

In light of these considerations, laryngeal epithelial premalignant lesion (LEPL) is an important stage in LSCC development. Thus, to further investigate LSCC pathogenesis and to avoid delayed diagnosis, upcoming studies aimed at defining a clear guideline to describe and treat these lesions are highly requested. To this aim, a strong interest has also been developed in finding both diagnostic and predictive biomarkers in LEPLs. Hu et al. discovered the diagnostic potential of both miR-10a-5p and miR-34c-5p in assisting histopathological classification of LEPL. In detail, miR-10a helped distinguish low-risk from high-risk lesions [24].

The mortality risk associated with SCC is strongly dependent on invasive and metastatic potential. Tuncturk et al. reported a proportional correlation between the upregulation of 9 miRNAs and the increased malignancy of the lesions [25]. Among these miRNAs, the expression of Hsa-miR-106b-3p was 3.01- and 7.31-fold higher in premalignant and malignant groups, respectively, compared to the benign group. On this basis, it has been proposed as a transformation biomarker [26]. In a recent study, Daquan et al. reported a decreased prognostic nutrition index value for LSCC compared to LEPL or laryngeal benign lesion (LBL). Moreover, poor nutritional index was correlated with higher risk of developing LSCC [27].

Furthermore, there is a great interest in understanding how long-noncoding RNA could affect tumor pathogenesis. For instance, Lan et al. clarified the role of LINC00886, which was downregulated by promoter hypermethylation with a consequent effect of stimulating epithelial-mesenchymal transition (EMT), proliferation, migration, and invasion processes. In addition, decreased LINC00886 expression was correlated with pathological differentiation grade and metastasis occurrence [28].

In this context, an integrated clinical, pathologic, and genetic evaluation of LSCC is required for the correct assessment of invasive grading, as well as for the definition of a suitable surgical and oncologic treatment. Immunohistochemistry has a huge relevance in LSCC for the need to take advantage of prognostic factors correlating with cancer evolution and histopathological degree of the lesion.

## 3. Molecular Markers for Clinical Management of LSCC

### 3.1. Mutated Genes and Abnormal Protein Expression in LSCC

Numerous LSCC-related genetic alterations have been described to date, though the actual molecular effects that specifically drive the onset and progression of this neoplasm remain almost uncertain. Therefore, the understanding of the downstream molecular changes of such aberrations could help in clarifying the mechanisms of underlying cancer progression and, above all, it could direct the identification of specific biological targets for the development of new treatment strategies. It is very important to point out that over half of LSCC patients with advanced disease (stage III or IV) at the time of diagnosis experience recurrence or the onset of distant metastasis. For this reason, there is a clinical need to identify novel biomarkers for both early diagnosis and effective timely treatment to improve patients’ outcomes. In depth characterization of LSCC-related molecular profiles represents the most promising approach to achieve this goal, and next generation sequencing (NGS) is a reliable procedure to elucidate the mutational landscape of LSCC [29].

Based on the recently identified mutations in LSCC, the major pathologic pathways implicated in its tumorigenesis include the dysregulation of some key processes, such as cellular survival and proliferation (Tp53 and EGFR), cell-cycle control (CDKN1A), and cellular differentiation (NOTCH1) pathways [30]. In this context, we tried to focus our attention on widely studied and recently identified mutations and abnormal protein expression, describing them for the different functions, modes of action, and clinical roles as prognostic biomarkers.

*p53.* p53 is a key regulator of genomic stability and acts as a tumor suppressor protein. Numerous studies have identified *TP53* mutations in HNSCC with an incidence ranging from 50% and 80% [31,32], and a strong correlation with poor survival, which was even greater in the case of disruptive mutations [31]. Poeta et al. observed *TP53* mutations in 224 of 420 tumor patients (53.3%) which also showed decreased overall survival (OS) compared with the wild-type ones [31]. Moreover, p53 expression, together with Mouse double minute 2 homolog expression (MDM2), was positively correlated with advanced stage of LSCC by Chrysovergis et al., as demonstrated by immunostaining of p53 and MDM2 in the 50 LSCC patients included in their study. The results showed p53 overexpression in 32% LSCC patients and a stronger MDM2 expression in 44% cases [33]. *TP53* gene mutations have been also assumed to affect the response to radiotherapy and drive both cell differentiation and neck LNM, although no correlation with LSCC clinical stages was observed [34,35]. Moreover, Pruneri and collaborators assessed the relationships between p63 expression and *TP53* gene status, suggesting that abnormal p63 levels may be involved in the early phases of laryngeal tumorigenesis irrespective of *TP53* mutational features [36]. In this study, both the prevalence and clinical implications of p63 immunoreactivity (IR) were evaluated together with mRNA expression in LSCCs. The authors analyzed 150 LSCC cases and results evidenced no significant association between p63 IR and patients’ survival. In detail, mRNA transcripts with (TA-p63) or without (ΔN-p63) trans activating properties were analyzed on p53-responsive genes. ΔN-p63 mRNA transcripts were detected in all the 23 tumors analyzed, whereas TA-p63 mRNA transcripts were absent in 5 cases. Moreover, *TP53* gene mutations were found in 24 of the 82 cases and p53 IR in 58 of 108, and neither event was associated with p63 IR. In addition, a downregulation of TA-p63 mRNA transcripts was observed in patients with T3–T4 tumors and advanced clinical stage [36]. Of note, another study analyzed the genetic changes associated with the transformation-progression of 94 tissue samples by multiple ligation-dependent probe amplification (MLPA). As a result, it showed a copy number variation for 37 genes. Among these, the most common were *TP53*, *IL1A*, and *RB1* losses and *STK11* gain, all associated with metastatic primary tumors, while the LNM were characterized by *LMNA*, *RECQL4* and *IGF1R* losses, and both *N33* and *CDKN2D* gains [37].

*Bcl-2.* Bcl-2 family members are involved in the regulation of apoptotic cell death, acting as either anti-apoptotic (Bcl-2 and Bcl-xL) or pro-apoptotic (Bax and Bak) mediators, or, additionally, working dually as anti-apoptotic or pro-apoptotic isoforms (Bcl-x, Bcl2L12) by alternative splicing. Together with p53, Bcl-2 plays a central role in the regulation of apoptosis. It has been proven that p53/Bcl-2 co-expression was significantly associated with poor differentiation, tumor extension, LNM, and advanced clinical stage of LSCC [38]. In a recent study, 78 primary LSCC patients were analyzed, and the Kaplan–Meier survival curves showed advanced LSCC patients with BCL2L12-positive tumors characterized by significantly longer OS time and a lower risk of death. These data suggested that BCL2L12 protein expression could be used as a favorable prognostic tissue biomarker in patients with advanced primary LSCC. However, Bcl-2 and Bax levels were not correlated with primary LSCC patients’ prognosis [39]. Another recent study showed high Bcl-2 protein expression levels in 42% LSCC tissue sections, whereas the remaining cases exposed a low expression. In depth evaluation displayed that Bcl-2 overexpression correlated with aggressive phenotype and anatomic localization, as well as with negative response to radiotherapy [40]. The prognostic potential of the Bax/Bcl2 ratio in LSCC was also analyzed by real-time qPCR, showing decreased risk of relapse and overall good prognostic outcome for patients with high Bax/Bcl2 ratio, with special reference to N0 patients, predicting for them longer DFS and OS independently from histological grade, tumor size, and TNM stage, as evidenced by Kaplan–Meier survival analysis [41].

*Cyclin D1.* Cell cycle-related proteins have also been widely investigated in LSCC to identify molecular markers for early diagnosis and prognosis and to find new therapeutic targets. Recent insights revealed a significantly higher positivity for cyclin D1 immunostaining in LSCC patients compared with both subjects suffering from laryngeal dysplasia and healthy individuals. In detail, this protein was identified as a highly sensitive (81.2%) and specific (83.9%) marker of LSCC compared to laryngeal dysplasia, and a highly sensitive (81.2%) and lower specific (41.4%) marker in comparison with healthy laryngeal mucosa. FGF3 and p16 levels were also significantly upregulated in LSCC patients compared with both laryngeal dysplasia and healthy mucosa groups. Moreover, both cyclin D1 and p21 positive immunostaining strongly correlated with the occurrence of regional LNM [42].

*p21 and p27.* Among other cell cycle regulators, the prognostic significance of p21 and p27 protein expression in LSCC was investigated for the first time by Pruneri and colleagues in 1999, when a role of independent predictor marker for prognosis was identified for p27 [43]. Almost concurrently, reduced p27 expression in LSCC was correlated with aggressive behavior, advanced clinical stage, and metastatic disease [44,45]. More recently, increased p21 expression has been significantly associated with higher cyclin D, cyclin E, and Ki67 levels, while p27 upregulation was correlated with p53 accumulation and increased proliferation, thus suggesting the establishment of a protective mechanism against the inhibitory effect exerted by these proteins on cell cycle progression [46].

*EGFR.* One of the molecules involved in the occurrence of LSCC is epidermal growth factor receptor (EGFR). The EGFR pathway induces cell transformation through autocrine overproduction of epidermal growth factor/transforming growth factor α (EGF/TGFα). EGFR overexpression by gene amplification can alter the transcriptional mechanism of survival. The role of combined EGFR/anaplastic lymphoma kinase (ALK) expression as molecular marker was analyzed in 25 primary LSCC patients by immunohistochemistry (IHC). In detail, EGFR overexpression, observed in 68% of cases, was correlated with tumor aggressiveness (*p* = 0.049), while ALK low expression in 92% was associated with stage (*p* = 0.048). A biphasic EGFR protein expression pattern was observed for some LSCC patients, whereas ALK levels were stable in all cases. In conclusion, EGFR overexpression is frequently observed in LSCC combined with low ALK levels, thus suggesting possible targeted therapeutic strategies for patients with a high EGFR/ALK ratio [47]. In recent work, a total of 38 LSCC cases were analyzed by IHC paying particular attention to age, gender, histological grade, depth of invasion (pT), lymph node metastasis (pN), and tumor stage. Metastases were not diagnosed (pM0) in any of the present cases. The results confirmed a significant increase of EGFR values, mostly in poorly differentiated carcinomas compared with moderately and well differentiated ones [48]. Furthermore, the correlation between *EGFR*, *cyclin D1*, and *KRAS* in LSCC and how the three genes play a synergistic role in the onset and development of this neoplasm has also been reported. Expression levels in LSCC tissues were analyzed by IHC and correlated with clinical features by statistical analysis. The results indicated that EGFR, cyclin D1, and KRAS levels are closely associated with clinical stage and metastasis or recurrence after treatment. In detail, the expression of the three genes in LSCC tissues increased compared to vocal cord polyp tissues and acted synergistically to promote the onset and development of LSCC. The Kaplan–Meier method, used to evaluate the mean survival time and survival rate of patients, showed a significant decrease of these parameters for EGFR, cyclin D1, and KRAS high-expressing groups, influencing prognosis as well as age, smoking, and alcoholism, that were considered relative risk factors by multivariate analysis of prognosis using the Cox regression model, while clinical stage and response to treatment were independent risk factors [49].

*E-cadherin.* EMT refers to a process whereby the adhesive polarity of epithelial cancer cells dissipates and changes to mesenchymal cells. Cadherin switching, consisting in E-cadherin decrease and simultaneous N-cadherin raise, is a feature of EMT in numerous types of malignant tumors, including HNSCC, where this process often correlates with the occurrence of LNM [50]. E-cadherin expression and its correlation with both clinic-pathological features and LSCC prognosis was assessed in 75 patients by IHC. The low expression rate of E-cadherin was reported in about 50.7% versus 83.2% cases, as demonstrated by Larizadeh et al. [51]. Meanwhile, a significant association between reduced E-cadherin expression and tumors of the supraglottic region, poor tumor differentiation, LNM, advanced T-stage, and TNM stage was identified [52]. A similar observation was reported by Nardi et al. who evaluated by IHC analysis the expression of E-cadherin and β-catenin in 52 LSCC tissue samples. The authors showed that E-cadherin and β-catenin decrease lead to a greater local aggression and cervical metastases occurrence [53]. In a recent retrospective study, EMT-associated markers’ expression (E-cadherin, N-cadherin, β-catenin, and ZEB2) was analyzed in 76 stage I–IVa LSCC patients treated with surgery, with and without LNM, using IHC analysis. In particular, the clinic-pathological significance of E-cadherin/N-cadherin, E-cadherin/β-catenin, and E-cadherin/ZEB2 co-expression in LSCC was assessed. ZEB2 is a transcriptional repressor which induces EMT by suppressing E-cadherin expression, thus contributing to the invasiveness of malignant tumors. Therefore, it has been considered a predictor of poor prognosis in numerous types of cancer, including HNSCC. The results of the current study also evidenced the role of ZEB2 expression as a critical factor in predicting the prognosis of LSCC. N-cadherin was also indicated as an EMT biomarker based on its association with oncogenesis, development, and metastasis in LSCC [54].

*CTNNA2 and CTNNA3.* Catenin Alpha 2 and 3 (CTNNA2 and CTNNA3) were found mutated in 15% of LSCC tissues analyzed by exome sequencing by Fanjul-Fernández and colleagues, who hypothesized a tumor suppressor function for these genes. CTNNA2 and CTNNA3 are key proteins of the adherents’ junctional complex in epithelial cells. To evaluate the impact of *CTNNA2* and *CTNNA3* mutations in the clinical outcome of LSCC patients, the authors examined the available clinical data of 86 patients, including 7 with the mutated isoform of at least one of these genes. As a result, the mutations were associated with a worse clinical prognosis. Furthermore, they evaluated cell invasion under conditions of loss or gain of function of these α-catenins, supporting the hypothesis that *CTNNA2* and *CTNNA3* are tumor suppressor genes and their genetic inactivation endows HNSCC cells with migration and invasion advantages, thus contributing to LSCC progression [55].

*NOTCH.* NOTCH signaling pathway is highly conserved evolutionarily, playing central roles in embryonic development and adult life, as well as regulating tumor biology in a context-dependent manner. To better understand the alterations of NOTCH signaling pathway, Sun et al. performed DNA copy number, methylation, expression, and mutation analysis from a cohort of 44 HNSCC tumors and 25 normal mucosae [56]. In detail, they provided robust evidence about the leading activation of NOTCH pathway because of ligand receptor copy number increase in HNSCC subsets with wild type *NOTCH1* and, against, a deficiency of NOTCH pathway in correspondence of loss of function mutations. These results suggest the relevance of NOTCH-targeting therapies in *NOTCH1* wild type tumors [56]. In fact, *NOTCH1* activity is contextual, playing a bimodal role as tumor suppressor or oncogene [57]. Regarding the biomarker properties of this gene, a study showed that NOTCH1 expression in the LSCC tissues of 106 patients was significantly associated with clinical stage and LNM, supporting the role of NOTCH1 in the malignant progression of human LSCC [58]. Meng-Yuan et al. analyzed NOTCH1 expression in 55 LSCC samples (18 cases with metastasis, 37 without). They performed a tissue microarray of 40 LSCC cases (5 cases with metastasis and 35 without) and both quantum dot immunohistochemistry (QD-IHC) and conventional IHC analysis to comprehensively confirm NOTCH1 expression levels in LSCC tissues. Moreover, the authors silenced *NOTCH1* in human laryngeal carcinoma HEp-2 cell line to elucidate the downstream effects on LSCC cell proliferation, apoptosis, migration, and invasion, demonstrating the contribution of NOTCH1 to the last two processes. Nevertheless, further studies are warranted to determine the molecular mechanisms underlying the role of NOTCH1 in LSCC invasion and metastasis [59]. There are also other mutations capable of activating crucial components of the NOTCH signaling pathway, including *NOTCH2* or *3* receptors, which may result in LSCC. As revealed by QD-IHC, an upregulation of NOTCH2 expression was observed in LSCC tissues compared with vocal cord polyps and a further upregulation was recorded for LSCC tissues from patients with LNM compared to the non-metastatic ones. The analysis was performed on 23 LSCC samples with LNM, 72 LSCC samples without, and 31 samples from vocal cord polyps. The authors also demonstrated that NOTCH2 signaling contributes to cell growth, survival, and metastasis in LSCC [60].

*NAT1 and NAT2.* Other cancer-related polymorphisms affect enzymes involved in the biotransformation of tumorigenic components of tobacco, such as N-Acetyltransferase 1 (NAT1) and 2 (NAT2), which actively participate in the metabolic activation of aromatic and heterocyclic amines to electrophilic intermediates. The association of *NAT1* and *NAT2* genotypes to LSCC risk was analyzed between the end of the last millennium and the beginning of the new one, but no recent evidence is currently available. A significant, even if rare, overrepresentation of homozygous *NAT2* genotypes coding for rapid acetylation was reported for LSCC patients compared to healthy individuals [61]. Later, *NAT1* and *NAT2* polymorphisms were associated with an increased risk of LSCC, and a correlation between NAT1*10/*11 genotype frequency and tumor location was also observed [62].

*OGG1.* A notable mutation associated with an increased risk of laryngeal cancer involves the key enzyme OGG1 (the human 8-oxoguanine glycosylase 1), which performs excision repair versus 7, 8-dihydro-8-oxoguanine (8-oxoG), a mutagenic derivative resulting from the exposure to reactive oxygen species (ROS). The study was conducted on 210 patients with histologically confirmed laryngeal cancer and 210 cancer-free controls. The obtained data on gene environment interactions illustrated a positive association between smoking and LSCC for *OGG1* mutated patients: His699-700His, in case of light smokers, and Gln718Gln, Ala597Val, Glu707Lys, and His699-700His, in case of heavy smokers. In conclusion, both silent and missense mutations were found to accumulate in smokers and, particularly, His699-His700 silent mutation displayed an enhanced risk by ~9.0 fold to develop LSCC, thus suggesting a role in the risk prediction [63].

*PIK3CA, FGFR3, JAK3, MET, and FBXW7*. Mutations in *PIK3CA* (phosphatidylinositol-4,5-bisphosphate 3-kinase catalytic subunit alpha) and *FGFR3* (fibroblast growth factor receptor), two of the most important intracellular regulators of cell growth, have recently been detected in LSCC cases, through the use of next generation sequencing, followed by Sanger sequencing and/or qPCR validation. Interestingly, these mutations were also detected in the matched tissues of patients with antecedent laryngeal dysplasia. In contrast, mutations in *JAK3* (Janus kinase 3), *MET*, and *FBXW7* (F-Box and WD repeat domain containing 7) were found only in non-progressing dysplasia, but absent in progressing dysplasia and LSCC cases [29].

*PTEN. PTEN* is a tumor suppressor gene, member of the PI3K/AKT/PTEN/mTOR signaling pathway, which regulates many cellular functions including cell proliferation, protein synthesis, and survival. Decreased PTEN expression has been recently shown in LSCC with a specific correlation with the glottic region and a lower association with the supraglottic one. Mastronikolis et al. analyzed PTEN expression in 50 archival formalin-fixed, paraffin-embedded tissue samples of primary LSCC finding a downregulation in 56% of samples, while a strong or moderate expression in the remaining 44%, similarly to the normal-appearing laryngeal epithelia used as control group. PTEN downregulation was correlated with shorter OS and advanced tumor degree (grade II/III). Therefore, PTEN expression could be a useful prognostic marker of tumor aggressiveness [64].

*PARK7.* PARK7 (Parkinson protein 7), known as DJ1, acts as a positive regulator of androgen receptor-dependent transcription. An immunohistochemistry-based study correlated its overexpression with LSCC site, as well. Moreover, its upregulation was related to lymph nodal status, clinical stage, and patients’ outcomes [65]. It has been shown that DJ1 overexpression was negatively correlated with PTEN expression in the tumor tissues of patients with LSCC. siRNA targeting of DJ1 effectively upregulated PTEN expression, resulting in enhanced cell death, as well as decreased proliferation and invasion of HEp-2 and SNU-899 cells. These data suggested that DJ1-induced PTEN downregulation may be involved in the proliferation and invasion of LSCC [66].

*TrkB.* The altered expression and the occurrence of genetic mutations affecting tropomyosin-related kinase B receptor (TrkB), have been proven to play an essential role for tumor progression as observed by the increase of invasion, metastasis, angiogenesis, and resistance against therapeutic treatments. Even though the underlying mechanism remains unclear, TrkB was frequently overexpressed in highly metastatic laryngeal cancer cell lines, and in clinical laryngeal cancer samples, acting as a key regulator of the c-Src-mediated activation of PI3K/AKT signal pathway and driving EMT [67]. Therefore, TrkB could represent a therapeutic target for counteracting the metastatic process in LSCC. Interestingly, the therapeutic targeting of Trk has also been found to suppress tumor proliferation and enhance cisplatin activity in HNSCC [68].

Summarizing, the knowledge of LSCC molecular traits, i.e., the complex combination of genetic alterations and protein aberrations, could represent an important turning point for the identification of new molecular biomarkers, as well as of new treatment options for an upcoming clinical management of the neoplasm. A panel of molecular diagnostic and/or prognostic factors analyzed in the last years and reviewed in this work, are summarized in Table 1.

### 3.2. miRNA and lncRNA Signatures as Predictors of LSCC Spreading

#### 3.2.1. miRNAs as Tissue Biomarkers

Within the wide panel of molecular factors to be explored for their therapeutic and/or diagnostic potential, a primary role is played by the big family of non-coding RNAs, which arouse growing interest for their multiple biomedical applications.

miRNAs, known as a subset of small non-coding RNAs of approximately 22 nucleotides in length, post-transcriptionally regulate gene expression through binding target messenger RNAs (mRNAs), leading to their destabilization and degradation or translational repression [69]. To date, many studies demonstrated that a number of miRNAs are deregulated and work as oncogenes or tumor-suppressors in various cancer types including LSCC [69,70]. In addition, miRNA abnormalities have been found to correlate with metastasis occurrence in many malignancies or with other clinical-pathological parameters such as differentiation, T classification, or clinical grading, thus suggesting their valuable use as predictive biomarkers for early detection of tumor spreading [71]. In addition, these new potential biomarkers can predict occult metastasis and may substantially improve treatment strategies for LSCC patients, thereby increasing the 5-year OS after the surgery.

So far, it has been reported that many miRNAs are deregulated in the LSCC tissues of patients suffering from metastasis. Chen et al. showed different correlations between miR-141 and some clinical-pathological features [72]. For instance, they demonstrated significant downregulation of miR-141 in laryngeal cancer tissues from individuals with lymph node involvement (N+) compared to non-metastatic ones (N-), as well as for patients with high TNM stage and differentiation degree. The characterization of the pertaining molecular mechanism unveiled both in vitro and in vivo inhibition of EMT, LNM occurrence, and TGF-β pathway, through direct targeting of homeobox C6 (HOXC6), a well-known EMT regulator [72].

Continuing with the miRNAs recently characterized for their anti-metastatic activity, another study showed the decrease of miR-138 expression in LSCC tissues with distal metastases, with respect to non-metastatic specimens. Further investigation indicated that miR-138 suppresses LSCC cells’ ability to spread into adjacent tissues, regulating the invasion factor ZEB2 [73].

Afterwards, Zhao and colleagues found lowered miR-145 expression levels in N+ tissues compared to N- ones, while high levels were displayed in early T stages and well differentiated specimens. Moreover, low miR-145 levels were related to poor prognosis. In addition, the authors demonstrated the ability of this miRNA in suppressing LSCC cell proliferation and invasion, as well as in promoting the apoptosis through the direct targeting of MYO5A [74].

Similarly, miR-203 reduced expression in LSCC tissues was significantly associated with the occurrence of LNM, poor tumor differentiation, advanced T stage, and poor prognosis 5 years after diagnosis. Moreover, its ectopic expression in laryngeal cancer cells was demonstrated to inhibit cell growth and invasion, and to induce cell cycle arrest and apoptosis via regulating ASAP1 [75].

miR-204-5p was also down-modulated in N+ LSCC samples and in T3–T4 stages; functional analysis showed that miR-204-5p can attenuate cell proliferation, migration, invasion, and EMT processes in laryngeal cancer cells by targeting Forkhead box C1 (FOXC1). Additionally, it strongly inhibits in vivo tumor growth in mice xenografts injected with HEp-2 or TU-177 cells [76].

Anti-proliferative, anti-migratory, and anti-invasive in vitro properties were also demonstrated for miR-143-3p, which also suppressed in vivo tumor growth and showed significant low expression in LSCC N+ tissues compared to N- ones. Of note, the involved mechanism is the k-Ras/Raf/MEK/ERK signaling pathway, which mediates cell survival, proliferation, and metastatic transformation [77]. Another report revealed that miR-101 downregulation correlates with the occurrence of LNM, high tumor grading, and high clinical stages. Furthermore, it was also analyzed the 5-year OS, showing that low miR-101 levels correlated with poor LSCC prognosis. Additionally, it was confirmed that miR-101 inhibits both proliferation and migration and promotes cell cycle arrest and apoptosis via cyclin-dependent kinase 8 (CDK8) targeting in LSCC cells [78].

Another study analyzed miR-149 expression in laryngeal cancer tissues, finding a substantial downregulation in the N+ group, in highly differentiated tumors and in advanced clinical stages. Moreover, LSCC patients with decreaserad miR-149 expression exhibited a lower survival rate compared to the high-expressing ones. In vitro analysis showed that miR-149 suppresses cell growth and stimulates apoptosis as well [79].

The well-known tumor suppressor miR-34a [80] has been analyzed in laryngeal cancer tissues by Shen and colleagues and a significantly decreased expression was found in N+ patients. Moreover, miR-34a reduced expression was associated with shorter DFS time, and in vitro studies indicated that miR-34a inhibits cell growth by inducing cell cycle arrest and by decreasing the expression of the anti-apoptotic factor survivin [81].

Similarly, miR-195 inhibits cell proliferation, colony formation, migration, and invasion, promoting cell cycle arrest and apoptosis by directly targeting DCUN1D1, known as a squamous cell carcinoma-related oncogene. Clinically, miR-195 downregulation was observed in N+ LSCC tissues and Kaplan–Meier curves revealed that low miR-195 expression is statistically associated with reduced survival rate [82].

Li et al. showed that increased miR-744-3p levels in LSCC tissues are significantly correlated with LNM. Moreover, the authors suggested that miR-744-3p enhances the pro-metastatic ability of laryngeal cancer cells through programmed cell death 4 (PDCD4) and PTEN targeting, leading to the activation of matrix metallopeptidase 9 (MMP-9), which enables cancer cell migration [83].

A previous study demonstrated miR-21 upregulation in N+ LSCC tissues compared to N-. The same paper reported that miR-21 inhibitor suppressed in vitro proliferation and invasion, and induced cell cycle arrest and apoptosis of laryngeal cancer cells by decreasing Ras expression [84].

Another significantly overexpressed miRNA in N+ LSCC tissues is miR-129-5p, which is positively correlated with T classification. miR-129-5p is engaged in promoting cell proliferation and migration, and in suppressing apoptosis through modulating the key regulator of Wnt signaling pathway, adenomatous polyposis coli, APC [85].

Our research group performed a high-throughput microarray analysis for miRNA expression profiling in laryngeal cancer tissues. As a result, we defined a predictive miRNA signature for nodal metastases occurrence, including seven upregulations (miR-618, miR-542-5p, let7b, miR-135a, miR-20b, miR-324-3p, and miR-886-5p) and four downregulations (miR486-3p, miR-328, miR-376a, and miR-493) in the N+ group compared with N- ones [86]. A further characterization, by ROC curve analysis, displayed the strong potential of miR-449a as a predictive marker of nodal involvement. Through both gain and loss of function studies, we also proposed a possible mechanism of action via NOTCH1 and 2 direct targeting and functional in vitro assays demonstrated miR-449a-mediated inhibition of LSCC cell proliferation, migration, and invasion [87].

#### 3.2.2. miRNAs as Circulating Biomarkers

Even more interesting than tissue miRNAs, extracellular circulating miRNAs in biological fluids, including blood, serum, and saliva, are extremely stable and represent the new frontier of non-invasive biomarkers for many diseases including cancer, due to their high stability in virtue of the incorporation into exosomes, the binding to Argonaute proteins or to high-density lipoprotein [88]. In this frame, a number of studies revealed tumor-associated altered miRNA expression profiles, suggesting the identification of specific signatures endowed with a powerful diagnostic and prognostic potential [89]. However, despite of many reports about metastasis-related miRNA aberrations in laryngeal cancer tissues, the characterization of circulating miRNAs has not been fully elucidated due to several issues concerning the selection of a proper sampling method and the existence of different normalization strategies leading to different outputs, that strongly hinder their reliability for the practical clinical application. Among the most relevant papers, the study performed by Wang et al. demonstrated high expression of serum exosomal miR-21 in N+ LSCC patients, suggesting the availability as a potential prediction marker [90].

In another report, miR-155 expression in LSCC tissues and plasma was found significantly upregulated compared to non-cancerous specimens, and a close association was identified for both with higher tumor size, advanced stage, and LNM occurrence. Furthermore, ROC curve analysis showed a reasonable sensitivity and specificity as a potential early diagnostic LSCC biomarkers [91].

More recently, Cao et al. found that up-modulated serum miR-632 levels in LSCC patients are significantly associated with LNM stage, histological grade, and TNM stage. Moreover, Kaplan–Meier survival curves exhibited that the upregulation of serum miR-632 is correlated with short OS and DFS [92].

Furthermore, in the last few years, the interest of the scientific community in malnutritional- and cachexia-related potential cancer biomarkers has increased [93]. Powrózek et al. showed the prospective role of low of miR-130a expression levels in differentiating cachectic patients from moderately or mildly malnourished ones. Moreover, low miR130a levels were correlated with shorter OS compared to patients expressing higher level of this miRNA. The study included 70 head and neck cancer patients and 38 of them were affected by laryngeal cancer [94].

#### 3.2.3. lncRNAs as Tissue Biomarkers

Long noncoding RNAs (lncRNAs), a class of noncoding RNAs longer than 200 nucleotides in length, play crucial roles in a variety of cell signaling. In recent years, the aberrant expression of many lncRNAs in various tumors has been unveiled and a close association with tumorigenesis, metastasis, and tumor stage was identified for several neoplasms including LSCC. Hence, lncRNAs analysis stimulated great interest both as biomarkers and therapeutic targets for cancer [95]. To date, many lncRNA aberrations in LSCC patients suffering from LNM have been reported. For example, Qu et al. found upregulation of HOXA11 antisense RNA (HOXA11-AS) in N+ LSCC tissues, and the deregulation has been associated with poor prognosis. In addition, HOXA11-AS knockdown significantly suppressed cell growth, migration, and invasion of LSCC cells [96]. Another correlation with nodal metastases was identified for RGMB antisense RNA 1 (RGMB-AS1), whose high expression in LSCC tissues has been associated with advanced tumor stage and poor prognosis, as well. Further investigation revealed that *RGMB-AS1* silencing inhibits laryngeal cancer cell proliferation and invasion, and suppressed in vivo tumor growth, thorough miR-22 sponging and increasing NLRP3 expression [97]. Furthermore, Yang et al. showed that low expressed NF-κB interacting lncRNA (NKILA) in LSCC tissues significantly correlated with LNM, clinical stage, and poor prognosis. More in detail, NKILA functioned as a tumor suppressor by inhibiting laryngeal cancer cell viability and migration and promoting apoptosis through NF-κB modulation [98]. Wu et al. recorded high H19 expression in LSCC N+ tissues and correlated it with patients’ survival rate. Further studies revealed that H19 promotes LSCC cell proliferation, migration, and invasion through the regulation of both miR-148a-3p and DNMT1, known as a DNA methyltransferase enzyme [99]. Taurine-upregulated gene 1, TUG1, overexpression in N+ LSCC tissues was detected by Zhang et al., who demonstrated its oncogenic function by functional assays that showed increased cell proliferation, migration, and invasion, and apoptosis suppression in LSCC cells [100]. A significant association with nodal metastases, TNM stage, and pathological differentiation was also identified in the case of miR-155 host gene, MIR155HG, whose upregulation was detected in LSCC tissues. Additionally, it was confirmed that MIR155HG, induced by TGF-β, contributes to LSCC progression and EMT via regulating miR-155/SRY-related-HMG-box 10 (SOX10) axis [91]. Additionally, Wang et al. demonstrated that up-modulated nuclear paraspeckle assembly transcript 1, NEAT1, in LSCC tissues is correlated with LNM occurrence, advanced T grade (T3–4), and clinical stage. The oncogenic role of NEAT1 occurs by promoting LSCC cell progression through miR-107/CDK6 pathway regulation [101]. Similarly, high expression of suppressor of tumorigenicity 7 antisense RNA 1, ST7-AS1, was correlated with metastatic LSCC, advanced TNM stage, and poor prognosis. It plays an oncogenic role enhancing laryngeal cancer cell migration, tumor sphere formation, and tumor growth by regulating CARM1/Sox-2 axis [102]. Xiao et al. identified upregulated X inactive-specific transcript, XIST, expression in advanced stages T3–4 of LSCC tissues compared to T1–T2. In addition, the authors revealed that XIST knockdown arrest LSCC cell proliferation, migration, and invasion, and in vivo tumorigenesis, via regulation of miR-124/EZH2 axis [103].

#### 3.2.4. lncRNAs as Circulating Biomarkers

As well as miRNAs, metastasis-related circulating lncRNAs in LSCC patients still require deep investigation. Based on our knowledge, highly expressed serum HOTAIR in LSCC patients was significantly associated with LNM occurrence, advanced T classification, and clinical stage. The authors compared the exosomal content of both miR-21 and HOTAIR from 52 LSCC patients with 49 patients suffering from polyps of vocal cords. As a result, they found a powerful correlation between high ncRNAs levels and tumor occurrence, mainly when the two RNAs were analyzed in combination, as demonstrated by the huge increase of the area under the ROC curve when miR-21 and HOTAIR underwent combined examination. A 94.2% of sensitivity was also recorded in differentiating the tumor from benign lesion. Aside from a significant correlation for both RNAs with advanced stages, the authors also identified a strong connection with the occurrence of nodal metastases (p21, *p* = 0.0115; HOTAIR, *p* = 0.0003) [90].

Sun and colleagues also investigated the role of circulating lncRNAs in LSCC development and progression, in particular highlighting a significant increase of UCA1 serum levels in 90 LSCC patients compared to 90 healthy subjects. ROC curve analysis suggested that serum UCA1 may potentially serve as a diagnostic marker for laryngeal neoplasm (area under the curve was 0.8905, with a 95% confidence interval of 0.8408 to 0.9402, *p* < 0.0001). Apart from the diagnostic role, this work also analyzed the correlation between serum UCA1 expression and some clinical-pathological characteristics: the high serum levels of UCA1 were indicated to be slightly associated with distant metastasis and more strongly with OS rate (*p* = 0.032), underling the prognostic value of serum UCA1 for LSCC. Moreover, the effects of UCA1 were also studied on AMC-HN-8 human LSCC cell line. In detail, UCA1 overexpression promoted the proliferation, migration, and invasion of cancer cells, by activating the Wnt/β-catenin signaling pathway [104].

Small NF90-associated RNA (snaR) is another lncRNA upregulated in LSCC plasma samples compared to healthy controls. It acts as an oncogenic lncRNA whose plasma levels positively correlate with AJCC stages and with transforming growth factor beta (TGF-β1) in LSCC patients but not in healthy controls. In addition, follow-up study revealed a correlation between high snaR plasma levels and poor OS. In vitro experiments showed the influence of snaR overexpression on cancer cell proliferation, migration, and invasion through the upregulation of TGF-β1 in human LSCC cell lines. Therefore, high plasma snaR levels may serve as a potential biomarker, correlating with progression and predicting worse prognosis for LSCC patients [105].

All the miRNAs and lnRNAs herein mentioned are summarized in Table 2.

### 3.3. Circulating Tumor DNA and Circulating Tumor Cells: Prospective Search to Improve the Clinical Management of LSCC Patients

Cell-free DNA fragments originated from tumor cells—circulating tumor DNA (ctDNA)—can be detected in body fluids such as blood, saliva, urine, and cerebrospinal fluid. Together with circulating tumor cells (CTCs), they have been considered eligible non-invasive biomarkers for cancer [106,107]. In particular, ctDNA analysis through liquid biopsy can be useful for different clinical applications, including tumor diagnosis and prognosis, tumor progression, treatment response, and multidrug-resistance (MDR) monitoring, and minimal residual disease detection after surgery, overcoming the tissue biopsy limitations [108]. Most likely, ctDNA carries the same molecular alterations of the primary tumor, such as point-mutations [109], methylations [110,111,112], and either integrated or episomal viral sequences [113,114]. The mechanisms proposed for ctDNA release into the bloodstream are essentially three: leakage from (i) apoptotic or (ii) necrotic tumor cells, (iii) secretion by living tumor cells or circulating tumor cells through extracellular vesicles [115,116,117]. Compared with other well-known liquid biomarkers, ctDNA detection is more sensitive, specific, and accurate in defining tumor progression, prognosis, and response to targeted therapy. It can also be helpfully correlated to clinical features and, due to its half-life of less than 2 h, ctDNA can reflect the real time tumor status and/or treatment response. Moreover, ctDNA detection can also represent a valid tool for monitoring relapse more efficiently than through conventional follow-up [118].

Based on the multiple evidence outlined for HNSCC, clinical application of liquid biopsy in LSCC is currently under investigation with a good chance of success. For this reason, we believe it is vital to give an overview on the current knowledge about the use of liquid biopsy in HNSCCs, since it could provide useful insights for future research focusing on the specific case of LSCC. Many of these studies highlight the correlation between either ctDNA or CTCs and neoplastic disease progression, demonstrating that ctDNA amount in circulating compartment increases in patients with advanced and metastatic cancer, compared to the early-stage ones [119]. The detectable concentrations of ctDNA in healthy control people are usually around 1–5 ng/mL, while higher levels (5–1500 ng/mL) can be found in cancer patients. ctDNA can be isolated from ctDNA on the basis of somatic mutations; therefore, the normal fraction remains constant, while the increased amount of DNA levels in body fluids can be considered as a derivative of tumor burden [117,120]. Mazurek et al. demonstrated the potential prognostic role of ctDNA in oropharyngeal squamous cell carcinoma (OPSCC). They quantified the ctDNA plasma level from 200 HNSCC patients, comparing the results with a control group of 15 healthy individuals and verified high, though not significantly increased, ctDNA levels in HNSCC group. Interestingly, a significant difference between the OPSCC sub-group and the other HNSCC patients was observed. Moreover, they explored a possible correlation between ctDNA and a series of clinical-pathological parameters, showing higher ctDNA levels in N2–N3 patients than in N0–N1, and in stage IV patients compared to stage I–III ones. Otherwise, decreased levels of ctDNA were observed during cancer treatment, until a complete disappearance on the last day of therapy for all patients [121]. The relevance of ctDNA as non-invasive marker for OPSCC persistence/recurrence and prognosis was also supported by another study performed on a cohort of 22 OPSCC patients where, among 11 non-responders, 5 patients showed the simultaneous detection of somatic non-synonymous variants in both tumor tissue and plasma. The analysis of DFS carried out on ctDNA-positive and ctDNA-negative patients showed an average of 12.3 months for ctDNA-positive patients and 37.0 months for ctDNA-negative ones [122]. Likewise, Wang et al. examined the plasma and saliva of 47 HNSCC patients, detecting ctDNA in 96% of them. In detail, all the patients with early-stage disease and 95% of patients with late-stage disease presented measurable levels of ctDNA. When the analysis was performed based on tumor site, ctDNA was detected in 100% of patients with tumors of the oral cavity, larynx, and hypo-pharynx and in 91% of OPSCC patients. The excellent potential of ctDNA as HNSCC biomarker was confirmed by follow-up analysis of nine patients, three of which still showed considerable levels of ctDNA in saliva or plasma after surgery, before clinical or imaging evidence of disease relapse. In detail, for two patients with oral cavity cancer, ctDNA was detected in saliva and plasma 4 and 8 months after surgery, while the tumor recurrence was clinically relevant only 19 and 9 months after, respectively. As regards the LSCC patient, ctDNA was found in saliva 7 months after surgery, before the appearance of tumor recurrence. The remaining 6 patients with undetectable ctDNA during follow-up displayed better DFS [123]. A similar study carried out on 12 paired laryngeal cancer tissue and plasma samples supports the identification of ctDNA mutational footprint as predictive biomarker for recurrent disease. ctDNA derived from both non-recurrent and recurrent LSCC patients exhibited a different detection rate of somatic mutations. Moreover, a number of somatic variants detected only in ctDNA were assumed to derive from a small population of tumor cells endowed with a high apoptotic rate in heterogeneous cancer tissues of recurrent patients [124]. The study performed by Bettegowda and colleagues strengthens the emerging role of ctDNA for more efficient detection of advanced disease, rather than early-stage neoplasia. They tested the biomarker property of ctDNA on a cohort of 640 patients with various tumor types, including 10 HNSCC individuals. All patients were divided into two groups based on localized or metastatic disease. The latter group showed an increase by 27% of ctDNA levels compared with the localized disease one (82% vs. 55%). Unfortunately, the small number of HNSCC patients compromised the statistical significance of the analysis for the potential prognostic function of ctDNA in this group of patients, where ctDNA was detectable in 70% of patients [118].

Aside from the measurements of ctDNA amount, a great interest has also been aroused by the analysis of CTCs and ctDNA genetic and epigenetic alterations, some of which have been extensively examined for their biomarker properties. Aberrant DNA methylation was assessed for four genes—*p16* (*CDKN2A*), O6-methylgua-nine-DNA-methyltransferase (*MGMT*), glutathione S-transferase P1 (*GSTP1*), and death-associated protein kinase (*DAP-kinase*)—in both primary tumor and serum of 50 HNSCC patients. A significant correlation was identified between *DAP-kinase* promoter hypermethylation and both lymph-node involvement and advanced disease stage. The methylation pattern was the same in ctDNA and paired cancer tissue for 42% of HNSCC patients and, among these, 5 developed distant metastasis, while spreading occurred only in 1 patient negative for serum promoter hypermethylation. Moreover, 7 patients showing promoter hypermethylation in cancer tissue DNA and/or at ctDNA, were monitored for methylation analysis using another serum sample collected within the subsequent 6–72 months. Four patients negative for *DAP-kinase* promoter hypermethylation at both serum samples were clinically disease-free. One patient positive for gene methylation pattern in both tumor and serum DNA but negative in the second specimen collection did not present any disease recurrence after surgery. For the other two patients, the short follow-up did not allow any clear deduction [111]. Similarly, Schrock et al. demonstrated that methylation levels of short stature homeobox 2 (*SHOX2*) and septin 9 (*SEPT9*) quantified in plasmatic ctDNA of patients before treatment were significantly higher with respect to control group (AUC SEPT9 = 0.79, 95% CI, 0.74–0.85; AUC SHOX2 = 0.80, 95% CI, 0.75–0.85), while their evaluation during follow-up showed a significant correlation with vascular and lymphatic invasion, tumor staging, and LNM. In particular, the advantage of combining both biomarkers is the increase of specificity from 95% to 96%. A univariate Cox proportional hazards analysis confirmed the prognostic role of *SEPT9* methylation levels for an adverse OS, a higher risk of locoregional tumor recurrence, and the development of distant metastases. Moreover, patients exhibiting high methylation levels at an early phase, showed a higher death risk, thus suggesting for them a more intensive therapeutic plan and post-therapeutic surveillance. Hence, ctDNA methylation pattern might be used as a biomarker not only for molecular staging and prognosis but also for risk stratification and post-therapeutic monitoring of HNSCC patients [112].

The occurrence of ctDNA does not always appear together with CTCs, suggesting their potential distinct role. Nevertheless, the frequency of CTCs in HNSCC patients seems to increase along with the TNM stage, so that their detection in peripheral blood correlates with a less favorable prognosis. To date, several studies enrolled cancer patients with the aim to detect and quantify CTCs. Kawada et al. collected peripheral blood samples from 32 HNSCC patients and verified CTC occurrence in 29 of them. Although they found a significantly higher number of CTCs in patients with advanced stage, they did not show any correlation between CTC amount and clinical N classification. Furthermore, they observed a significant decrease of CTC count after treatment [125]. Another clinical application offered by early CTC detection is the possibility to improve HNSCC outcome, as demonstrated by Nichols et al. In detail, 15 patients with advanced HNSCC stage (III–IV) were investigated for CTC detection. In 6 of them, CTCs were successfully isolated (1–2 cells/7.5 mL of blood) and significantly associated with the occurrence of suspicious lung nodules. In the sample, 87% of patients with detectable CTCs were monitored during treatment and 80% of them showed disease relapse or progression, which, otherwise, was diagnosed only for 12.5% of patients with no detectable CTCs. In addition, the log-rank test used for progression-free survival (PFS) analysis suggested an improvement, even though not significant, of survival for CTC-negative patients, with respect to CTC-positive ones [126]. Within HNSCCs, only a few studies analyzed CTCs in LSCC patients. In their pilot study, Rizzo et al. examined pre- and post-operative CTCs in the peripheral blood of LSCC patients and evaluated their correlation with prognosis. They enrolled 8 LSCC patients at stage III and collected patients’ blood samples both before total/subtotal laryngectomy or bilateral neck lymph-node dissection and after surgery at 3, 6, and 12 months. The results revealed a worse prognosis in LSCC patients with high pre-operatively CTC count with respect to CTC negative patients who were disease-free during monitoring time. Patients with a decreased CTC number in the post-operative follow-up exhibited an improved treatment response [127].

Despite the advances in detection procedures and the evidence supporting the promising role of CTCs and ctDNA as LSCC biomarkers, the inherent studies are rather lagging behind the other solid tumors. Therefore, a future challenge would be to carry out further research about the application of minimally invasive liquid biopsy for LSCC management. This may provide a substantial improvement in clinical evaluation, allowing for easier tumor progression tracking, early detection of occult metastasis, and identification of therapeutic targets for the design of personalized treatments.

### 3.4. Major Epigenetic Changes as a Molecular Signature of LSCC

Epigenetic events occurring through cancer onset and progression can modify gene expression profile without inducing any change to the primary DNA sequence. DNA methylation, histone modifications, and non-coding RNA-mediated silencing are the main epigenetic processes whose impairment can destroy gene function, triggering malignant transformation. The identification of specific epigenetic profiles associated with certain cancer types or subtypes, represents a powerful resource to recognize the mechanisms underlying carcinogenesis and to identify new candidate biomarkers for diagnosis, disease monitoring, prognosis, and treatment response [128].

HNSCC carcinogenesis involves the accumulation of both genetic and epigenetic changes, whose crucial role in tumor transformation has been recently deepened, especially in DNA methylation process, due to their tumor/tissue specificity, potential reversibility, and the tight dependence on environmental factors [129]. DNA methylation refers to the covalent addition of a methyl group (CH3) at the 5-carbon of the cytosine ring, resulting in 5-methylcytosine (5 mC). This chemical modification is catalyzed by a family of enzymes known as DNA methyl transferases (DNMTs). Three DNMTs are required for maintenance (DNMT1) and establishment of new or de novo (DNMT3A, DNMT3B) DNA methylation patterns. The DNA methylation process occurs almost exclusively in the context of paired symmetrical methylation of CpG sites, where a cytosine is placed next to a guanidine. In human genomic DNA, CpG dinucleotides are focused in large clusters called CpG islands, usually associated with gene promoters [130]. In normal cells, CpG sites are heavily methylated, while CpG islands remain unmethylated, allowing the access to transcriptional factors and chromatin-associated proteins, thus promoting gene expression. Otherwise, CpG island hypermethylation negatively impacts on gene expression, resulting in gene silencing in a tissue-specific manner during early development or in differentiated tissues, and in human cancers too [131]. Hence, detection of aberrant methylation profile could represent a tumor-specific marker in different biological materials such as body fluids, exfoliated cells, and tumor tissues [132].

LSCC exhibits altered patterns of DNA methylation compared to normal tissues, displaying global hypermethylation of tumor suppressor genes, accompanied by transcriptional loss and hypomethylation of proto-oncogenes, with subsequent transcriptional reactivation. Tumor-related genes subjected to these phenomena show compromised genome stability, resulting in the alteration of cancer-related processes, such as cell cycle, DNA damage repair, apoptosis, angiogenesis, and cell–cell adhesion, thus supporting malignant transformation and spreading [133].

*p16* methylation is a frequent inactivation event in LSCC, detected in more than 80% of LSCC cases, mostly with high grading (G3) [134]. *p16* gene, or *CDKN2A* (cyclin-dependent kinase inhibitor 2A), is a tumor suppressor gene involved in cell cycle regulation, whose expression is significantly reduced in LSCC [135,136,137]. Temam et al. examined both exfoliated cells and tissue biopsy of 33 patients with untreated early-stage SCC of either supraglottic larynx or pharynx, for the assessment of CDKN2 promoter hypermethylation. They demonstrated that methylation is an early event in this neoplasm, suggesting a possible diagnostic function in patients screened for early laryngeal carcinoma [138].

Apart from the potential diagnostic role, accumulating evidence underlines an emerging prognostic function for promoter hypermethylation of several gene patterns frequently down-modulated in LSCC. PCDH17 (protocadherin 17) is a cell–cell adhesion tumor suppressor protein, whose loss can co-occur with tumor progression by enhancing proliferation and EMT [139]. *PCDH17* promoter methylation was evaluated in 16 cell lines and 81 primary LSCC tumors, suggesting that epigenetic dysregulation was one of the inactivation mechanisms leading to *PCDH17* transcriptional loss. In detail, the authors found a highly recurrent DNA hypermethylation pattern in 100% of cell lines and 40% of primary tumors, while epithelial cells from both buccal swabs of healthy donors and control tissues showed low DNA methylation levels. The observation was validated by DNA demethylation experiments, where decitabine treatment of two LSCC cell lines restored low levels of genes’ transcription. The authors suggest the possibility to consider recurrent *PCDH17* hypermethylation as a diagnostic and prognostic biomarker in LSCC, even if further studies enrolling more patients with different tumor grades and stages are needed [140]. Recent studies revealed the crucial role of *LZTS2* [141], *E-cadherin* [142], *DAPK*, *MGMT*, and *RASSF1* [143,144] as either independent or aggregate biomarkers, thus defining a possible epigenetic signature of LSCC [145,146,147]. *LZTS2* (leucine zipper tumor suppressor 2) mediates both carcinogenesis and cancer development, and its predictive value of poor prognosis and treatment response has been demonstrated also for nasopharyngeal carcinoma patients [148]. It is a cell cycle regulator frequently down-modulated in several tumors as a result of promoter hypermethylation. This event has also been analyzed in LSCC, where the *LZTS2* methylation profile has been correlated with cancer risk, progression, and prognosis. Shen et al. examined a cohort of 96 LSCC patients, showing that *LZTS2* promoter methylation was significantly higher compared to adjacent normal tissues. In particular, patients’ stratification based on smoking and clinical-pathological status, revealed that individuals with advanced clinical stage, as well as smokers and patients with LNM, displayed significantly greater promoter methylation levels. The potential diagnostic value of *LZTS2* promoter hypermethylation was estimated to have 94.7% sensitivity and 80.4% specificity (AUC = 0.920). A survival analysis was performed to evaluate a possible association between OS and *LZTS2* methylation status. The Kaplan–Meier analysis confirmed a significantly higher OS for LSCC patients with hypomethylated *LZTS2* promoter compared to hypermethylated patients. Univariate Cox proportional hazards analysis also showed a greater risk of death for the latter group of patients [141].

E-cadherin promoter is also hypermethylated in LSCC, mostly in association with aggressive tumor behavior [142,149]. E-cadherin (CDH1, ECAD) is involved in cell differentiation, migration, and extracellular signal transduction. Its downregulation in LSCC has been correlated with infiltrative growth, histological differentiation, LNM [150,151], and tumor localization [152,153]. Moreover, decreased E-cadherin expression was identified as a predictive marker of response to radiotherapy [154]. Likewise, *DAPK* (death-associated protein kinase), *MGMT* (O-(6)-methyl guanine-DNA methyl transfer-ase), *RASSF1* (Ras association domain-containing protein 1), and *ADAM23* (ADAM dis-integrin and metalloproteinase domain 23) are among the most frequently methylated tumor suppressor genes in LSCC tissues, showing high diagnostic and prognostic potential [143,144]. In particular, *ADAM23* promoter hypermethylation was significantly associated with T3 and T4 LSCC stages [142]. These genes can regulate many cancer-related processes, such as apoptosis, autophagy, metastasis, and DNA repair [155,156], thus contributing to tumor growth and progression. *E-cadherin*, *p16*, *MGMT*, and *DAPK* hypermethylation was analyzed in 253 laryngeal and hypopharyngeal cancer patients, highlighting the high frequency of this event, although no correlation was detected with mortality or second primary cancer occurrence [147]. Similarly, Pierini et al. analyzed promoter methylation status of *CDKN2A*, *MGMT*, *MLH1* (MutL homolog 1), and *DAPK* in 100 LSCC patients, correlating it with clinical features. They detected a significantly increased methylation of *p16* promoter in heavy smokers, while both *p16* and *MLH1* promoters were hypermethylated in patients with lymph node involvement, where these genes were also linked to cell migration, invasiveness, and aggressive phenotype. Conversely, an inverse correlation was found between *MLH1* hypermethylation and alcohol consumption [146].

The relevance of a specific epigenetic signature related to LSCC carcinogenesis was also found in two papers by Paluszczak and colleagues. They assessed the methylation frequency of 7 genes, including *DAPK*, *MGMT*, and *RASSF1*, in a cohort of patients with primary LSCC, recording a simultaneous hypermethylation of three or four genes in about 50% of the analyzed samples, also establishing a correlation with LNM [145]. Likewise, they analyzed the methylation status of several WNT cascade inhibitors (*DKK1*, *LKB1*, *PPP2R2B*, *RUNX3*, *SFRP1*, *SFRP2*, and *WIF-1*) in 26 LSCC cell lines and 28 primary LSCC samples, showing that 50% of cell lines and 70% of primary LSCC samples displayed promoter hypermethylation in at least four genes. Furthermore, they observed a significant difference in the frequency of *PPP2R2B* and *SFRP1* promoter methylation between cell lines derived from either primary or recurrent LSCC [157].

**Figure 1:**
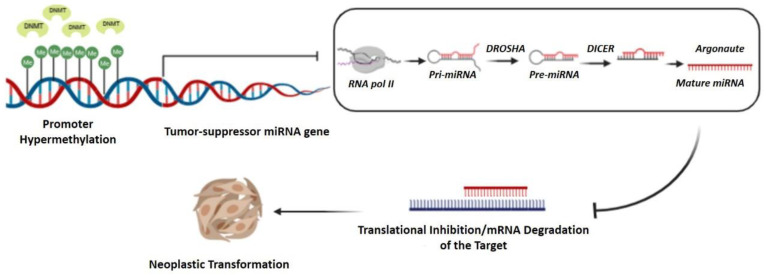
DNA methyltransferases (DNMT)-dependent regulation of miRNA expression. Promoter hypermethylation inhibits the expression of tumor suppressor genes, thus hindering their translational inhibitory function which results in neoplastic transformation.

**Figure 2:**
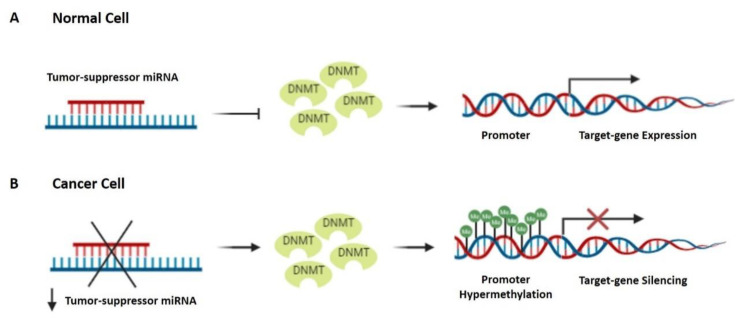
miRNA-dependent target-gene expression through DNMT regulation. (**A**) In normal cells, tumor suppressor miRNAs inhibit DNMT allowing target gene expression. (**B**) In cancer cells, the downregulation of tumor suppressor miRNAs activates DNMT-dependent silencing of target genes.

Similar to protein-encoding genes, miRNA expression can also be regulated by epigenetic modifications at the promoter region. In particular, tumor suppressor miRNAs are commonly silenced in cancer cells by DNA hypermethylation (Figure 1). Among the miRNAs epigenetically silenced in LSCC, miR-137 and miR-34a promoters’ hypermethylation are both associated with poor OS, disease progression, and metastasis, acting as poor prognostic factor for LSCC [157,158,159]. In addition to undergoing itself epigenetic regulation, miRNAs can also be able to control the epigenetic mechanisms (Figure 2). An example of mutual regulation in LSCC is represented by miR-184a-3p. Its silencing by the lncRNA H19 is positively correlated with LSCC progression through DNMT1 activation and subsequent hypermethylation of downstream suppressor genes, and significantly associated with tumor grade, differentiation, LNM, and clinical stage. Moreover, a Kaplan–Meier analysis confirmed a poorer OS rate for patients with high H19 expression [99].

On the other hand, the occurrence of oncogene activation through demethylation events is a less frequent process compared to the hypermethylation of tumor suppressor genes and generally occurs as a consequence of global hypomethylation, usually due to DNA methyl transferase dysregulation [160]. An example is represented by the regulation of acidic calcium-binding protein S100A4 overexpression, which has been correlated with DNA hypomethylation in several cancers, including LSCC [161]. Global *S100A4* promoter demethylation induces an increase of tumor invasiveness, as demonstrated in both LSCC tissue samples and HEp-2 cell line treated with DNA methyltransferase inhibitor. The aberrant DNA methylation was then reverted by transfecting HEp-2 cells with *S100A4* siRNA, thus confirming the hypothesis that transcriptional regulation of *S100A4* gene involves a DNA demethylation process [162].

## 4. Molecular Markers of Drug Resistance in LSCC

Drug resistance in anticancer treatment is a crucial issue and one of the leading reasons for poor response rates to chemotherapy. Despite the remarkable efforts to develop new effective molecules able to target oncogenic factors, there are still no successful chemotherapy agents for progressed cancers with metastases. So far, many studies have revealed that the acquisition of a resistant phenotype by cancer cells is mainly due to a number of factors, such as drug efflux, drug inactivation, drug target alterations, epigenetics, pharmacokinetic issues, EMT, DNA damage repair, and inhibition of cell death [163,164]. Therefore, understanding the molecular mechanisms underlying the circumvention of anticancer drugs’ cytotoxicity is essential to identify the best strategy to develop new pharmacological approaches for advanced stages of the diseases.

Recently, the molecular mechanisms of drug resistance in LSCC have been extensively investigated, focusing on the role played by non-coding RNA in both the acquisition and the maintenance of a resistant phenotype. Regarding the lncRNAs, Zhou et al. demonstrated that HOX transcript antisense RNA, HOTAIR, enhances chemo-resistance to cisplatin in LSCC cells through regulating miR-613 and SNAI2, a well-known EMT modulator. They also found that HOTAIR is significantly upregulated in LSCC tissues compared to normal healthy ones and that HOTAIR short hairpin RNA (shRNA) transfection suppresses tumor growth and in vivo EMT-related cisplatin resistance [165]. Conversely, p53-induced noncoding transcript, LINC-PINT, is lowly expressed in LSCC tissues with respect to the adjacent normal counterpart. Moreover, in vitro assays showed that LINC-PINT suppresses LSCC cell stemness and cisplatin resistance by directly targeting miR-425-5p, which regulates PTCH1, a Hedgehog pathway-related protein [166]. Another report showed FOXD2-AS1 overexpression in LSCC tissues, and this deregulation was significantly associated with poor prognosis. Additional studies exhibited that FOXD2-AS1 maintains laryngeal cancer cell stemness and reduces response to cisplatin. In addition, FOXD2-AS1 acts as a scaffold for signal transducer and activator of transcription 3 (STAT3) and protein arginine methyltransferase 5 (PRMT5), enhancing STAT3 transcriptional activity [167]. Yuan et al. demonstrated that actin filament-associated protein 1 antisense RNA1 (AFAP1-AS1), which is highly expressed in LSCC tissues compared to adjacent normal counterpart, promotes LSCC cell stemness and enhances cisplatin resistance through the direct targeting of miR-320a which, in turn, negatively regulates RBPJ, a crucial factor in Notch signaling pathway [168]. The highly conserved metastasis-associated lung adenocarcinoma transcript 1, MALAT1, is significantly upregulated in LSCC tissues, as well, and its deregulation was associated with poor 5-year survival rate and enhanced chemo-resistance of LSCC cells to cisplatin, 5-fluorouracil, paclitaxel, and vincristine by promoting cell proliferation, migration, invasion, and EMT, and apoptosis suppression [169].

In terms of chemo-resistance-associated miRNAs, Liu et al. showed that miR-125a is downregulated in LSCC tissues and HEp-2 laryngeal cancer stem cells (HEp-2-CSCs) as compared to peritumor non-cancerous tissues. Gain of function analyses demonstrated that miR-125a significantly enhances the sensitivity of HEp-2-CSCs to cisplatin through the direct targeting of hematopoietic cell-specific protein 1-associated protein X-1 (HAX-1), which plays an anti-apoptotic role by suppressing mitochondrial apoptosis pathways. Furthermore, transfection of miR-125a mimic attenuates multidrug resistance of HEp-2-CSCs to vincristine, etoposide, and doxorubicin [170]. Tain et al. found low miR-26b expression in cisplatin resistant HEp-2 (HEp-2/R) cells compared to HEp-2 wild-type model. Additional investigation indicated that ectopic expression of miR-26b suppresses cisplatin resistance in HEp-2/R by directly targeting activating transcription factor 2 (ATF2). The last is a key regulator of DNA repair, so its inhibition promotes the activation of Bcl-xL-mediated mitochondrial apoptotic pathway [171]. Other in vitro experiments performed on vincristine-resistant HEp-2v cells suggest increased cisplatin resistance and miR-133a downregulation relative to HEp-2 wild type model. Moreover, ectopic miR-133a restoration significantly enhanced the drug sensitivity of HEp-2v to cisplatin by targeting copper transporter P-type adenosine triphosphatase (ATP7B), known to be correlated with tumor cell sensitivity to cisplatin [172]. More recently, Lin et al. showed that miR-936 is reduced in LSCC tissues and associated with advanced T grade, poor differentiation, and LNM occurrence. In addition, miR-936 inhibits LSCC cell proliferation, migration, and invasion and improves drug sensitivity to doxorubicin and cisplatin through the direct targeting of GPR78, an orphan G-protein coupled receptor [173].

**Figure 3:**
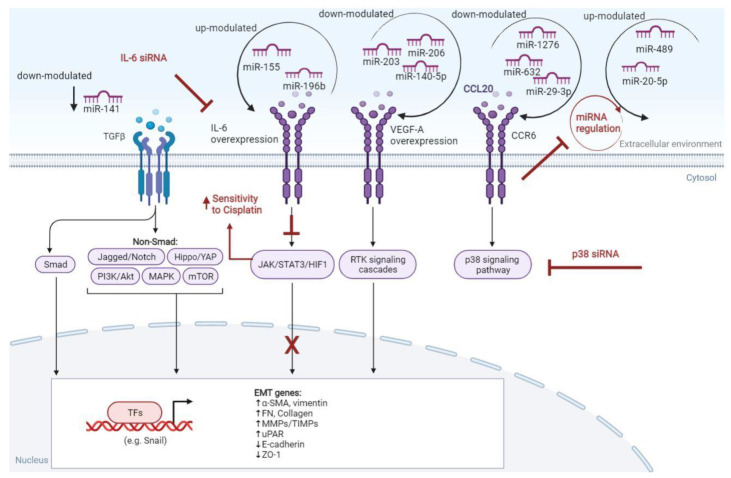
Schematic representation of multilevel interactions between extracellular molecular mediators of LSCC. Both extrinsic and intrinsic pathways contribute to triggering immune response and inflammation in LSCC, promoting EMT process, cell proliferation, motility, and chemo-resistance. The delivery of siRNA towards specific targets can improve the drug response or opportunely alter the miRNA expression profile improving patients’ prognosis.

Fu et al. revealed the up-modulation of interleukin-6 (IL-6), STAT3 and hypoxia-inducible factor 1 (HIF1) at both mRNA and protein levels in HEp-2-CSCs compared to HEp-2 wild type and LSCC tissues. Moreover, *IL-6* knockdown by siRNA technology in HEp-2-CSCs decreased not only IL-6 expression levels, but STAT3 and HIF1 as well, leading to the suppression of LSCC proliferation, colony formation, invasion, and tumor growth, and inducing apoptotic cell death. Importantly, the combination of *IL-6* siRNA and cisplatin significantly enhanced the suppression of all these parameters in HEp-2-CSCs, indicating that *IL-6* knockdown can increase drug efficacy in LSCC by modulating IL-6/STAT3/HIF1 pathway (Figure 3) [174].

It was also found that procollagen-lysine 2-oxoglutarate 5-dioxygenase 2 (PLOD2) improves cisplatin resistance in LSCC cells through the activation of Wnt signaling pathway. In addition, it enhances stem cell characteristics and its overexpression in LSCC tissues correlates to advanced cTNM and poor patients’ prognosis [175]. Of note, the tumor suppressor TIP30 was downregulated in drug-selected laryngeal cancer cells (DSCs) in comparison with wild type HEp-2, and its decreased levels were significantly associated with advanced T stage and poor prognosis in LSCC patients. Functionally, the loss of TIP30 contributes to self-renewal, proliferation, and drug resistance of laryngeal cancer cells through regulating AKT/glycogen synthase kinase-3 β (GSK-3 β)/β-catenin signaling. Hence, TIP30 suppresses self-renewal and cisplatin resistance in vitro. Moreover, in vivo studies revealed that it inhibits both tumorigenesis and cisplatin chemo-resistance in LSCC cells subcutaneously transplanted in nude mice [176]. Li et al. found that vincristine (VCR)-resistant HEp-2 (HEp-2/VCR) cells are chemo-refractory to VCR, methotrexate, cisplatin, and 5-fluorouracil, in comparison with HEp-2 wild type cells. Functional analyses revealed that the expression levels of proto-oncogene c-fos and multiple drug resistance 1 (MDR1) are increased in HEp-2/VCR. Moreover, it was demonstrated that c-fos enhances efflux capability of cytotoxic drugs in LSCC cells by promoting MDR1 expression, leading to the development of drug resistance [177]. A significant overexpression of both Wnt1-inducible signaling protein 1 (WISP1)—downstream target of Wnt/β-catenin signaling—and glucose transporter 1 (GLUT1) was observed in LSCC tissues. Additional in vitro studies showed that WISP1 enhances drug resistance and inhibits ataxia-telangiectasia-mutated (ATM)-mediated DNA damage response in laryngeal cancer cells treated with cisplatin, by activating Yes-associated protein 1 (YAP1)/TEA domain transcription factor 1 (TEAD1) signaling, that contributes to GLUT1 upregulation [178]. Interestingly, Xu et al. explored for the first time the precise role played by the apoptosis inhibitor survivin on hypoxia-induced MDR of LSCC, showing that its knockdown could partly reverse this phenomenon due to survivin’s ability to prevent chemotherapeutic drug-induced apoptosis. Therefore, targeting the survivin signal pathway has been suggested as a possible approach for reversing multidrug resistance in LSCC [179].

## 5. Microenvironment-Derived Inflammatory Markers and Immune-Related Factors in LSCC

As in many other solid malignancies, laryngeal cancer progression and invasion is strongly influenced by a pro-inflammatory tumor microenvironment (TME) [180]. Indeed, there is a functional interaction between inflammation, innate immunity, and cancer, re-siding on the physiologic and pathologic processes triggered by response to injury that involve multiple chemical factors able to activate tissue mast cells, directing migration of leukocytes (neutrophils, monocytes and eosinophils) from the circulatory bloodstream to the damaged tissue. In 1986, Dvorak HF started from the concept, already known since the 1970s, that there is a close interconnection between solid tumor stroma generation, wound healing, and chronic inflammation to demonstrate that these phenomena shared key molecular factors [181]. In detail, for all of them, what stands out is the boosted vascular permeability to plasma and plasma proteins because of increased levels of vascular endothelial growth factor (VEGF-A). Moreover, plasma extravasation can trigger the extravascular deposition of fibrin, which, in turn, provides a favorable substrate for the attachment and migration of tumor cells, also playing a key role in the generation of new blood vessels and connective tissue stroma. Therefore, VEGF-A levels being constantly high, tumors may continually initiate new healing activity, thus growing and invading surrounding normal tissues. In 2016, Xu and colleagues demonstrated VEGF-A suppression by miR-203 in LSCC, following the discovery of significant miR-203 down-regulation in LSCC tissues [182]. A negative relationship between miR-203 and VEGF-A was also identified in clinical tissue samples, as well as between miR-203 and cyclooxygenase-2 (COX-2), which was identified and validated in the same study as another miR-203 target. Moreover, through functional studies, it was demonstrated that a tumor suppressor function is played by miR-203 by inhibiting proliferation, migration, and invasion of HEp-2 cells. COX-2, commonly upregulated at the inflammatory sites and specifically overexpressed in HNSCC, has a consolidated use as an inflammatory marker in both stroma and tumor [183,184]. Starting from a bioinformatics analysis, a recent study revealed and subsequently validated, by luciferase reporter assay, the direct VEGF-A targeting by miR-140-5p, also proving its effect in counteracting cell proliferation, migration, invasion, and angiogenesis in LSCC, using a transfection approach based on miR-140-5p knocking-down or over-expression [185]. Another validated VEGF-A modulator is miR-206, whose transfection in laryngeal cancer cells was used to investigate proliferation, apoptosis, migration, and invasion, demonstrating its tumor suppressor function [186]. Moreover, an inverse correlation was found between miR-206 levels and T grade, LNM, and clinical stage of LSCC (Figure 3).

In the last twenty years, EMT has gradually emerged as a new concern relating to cancer cell invasiveness and has been increasingly studied, highlighting the main difference between migration of normal keratinocytes and transformed epithelial cells. EMT occurs exclusively in cancer cells and is characterized by adherents’ junctions breaking and epithelial markers’ (i.e., cytokeratins and E-cadherin) reduced levels, accompanied by mesenchymal markers’ (i.e., fibronectin, N-cadherin, and Vimentin) upregulation [187]. In addition to the leading role played by all these changes in gaining an invasive fibroblastoid phenotype, EMT can also lead to the modulation of other features of cancer progression, such as the decreased susceptibility of mesenchymal cells to immune clearance due to reduced physical connection between immune and target cells, which acquire a characteristic phenotype known as mesenchymal immune evasion (MIE) [120]. A major player of this condition is the transforming growth factor-beta (TGF-β), which can change the TME in the direction of a tumor-supportive environment through the enhancement of immuno-suppression by regulatory T cells (T-regs) activation [120]. Today, it is evident that cell proliferation can cause cancer onset when sustained by a microenvironment rich of pro-inflammatory cells and mediators, growth factors, activated stroma, and DNA-damage-promoting agents, including cytokines, chemokines, cyclooxygenase-2, prostaglandins, fibroblast growth factor (FGF), MMPs, and the already mentioned VEGF [180]. Among the pro-inflammatory cytokines, IL-6, together with IL-1 and TNF-α, is one of the most relevant promoters of acute inflammatory response, also capable of reinforcing chronic phase inflammation, providing the favorable environment for tumor growth. IL-6 is secreted by B and T lymphocytes, macrophages, fibroblasts, keratinocytes, as well as by cancer cells, controlling both proliferation and apoptosis processes [188]. IL-6 levels have been significantly correlated with tumor invasion, severity, spreading, and chemo-resistance depending on the cellular downstream pathways after binding to its specific receptor [189]. The latter activates the JAK/STAT axis, which in turn enhances metastasis via EMT induction, increases motility via focal adhesion kinase (FAK), and improves tumor invasion through the activation of VEGF and rho [190]. The analysis of pre-operative serum levels of IL-6 and IL-8 as prognostic variables in a cohort of 92 LSCC patients revealed significantly higher levels in patients compared to healthy controls (*p* < 0.0001) [191]. Moreover, IL-6 was associated to the occurrence of LNM (*p* < 0.001), T classification (*p* < 0.001), and clinical stage (*p* = 0.001), also showing excellent characteristics as independent predictor of LSCC-specific progression-free (*p* = 0.049) and OS (*p* = 0.040). The same study associated increased serum IL-6 levels to shorter OS and PFS (*p* < 0.05). The relationship between IL-6 serum levels and both severity and LSCC extent was also analyzed by other researchers, confirming the higher levels in tumor patients (*p* = 0.0001) and the significant correlation with advanced stage (*p* < 0.0001), as well as with the occurrence of metastasis (*p* = 0.024) and local tumor spread (T) *p* < 0.0001) [192]. miRNA-mediated modulation of the IL-6-signaling pathway has been extensively investigated in several neoplasms, such as breast [193], colorectal [194], lung [195], and prostate cancer [196] and multiple myeloma [197], but it is still not explored in LSCC. However, a recent investigation on LSCC tissues from 63 patients revealed a significant inverse correlation between miR-155, which was overexpressed in tumor tissues compared to control mucosa and SOCS1 protein, which, in turn, inversely correlated with STAT3 protein expression in the same samples [198]. Interestingly, miR-155 levels significantly correlated with stage and degree of cell differentiation (*p* = 0.014 and 0.021, respectively). Similar results were also found by the same authors for SOCS2 regulation by miR-196. In this case, the analysis of the possible connection with clinical-pathological parameters revealed that high miR-196b levels correlated also with cervical nodal metastases [199]. Further evidence of miRNA intervention in EMT process is the targeting of HOXC6 by miR-141, whose downregulation in LSCC was associated with the counteraction of TGF-β signaling pathway (Figure 3) [72]. Moreover, the ectopic expression of miR-141, as well as HOXC6 silencing in AMC-HN-8 laryngeal cancer cells, hindered the expression of TGF-β1, Smad3, Vimentin, and Snail and enhanced E-cadherin protein levels. Moreover, miR-205 and miR-375 are among miRNAs involved in LSCC progression via EMT regulation [200]. As regards lncRNAs participation in LSCC-related EMT, at the moment, we still have little experimental evidence. In particular, the miR-155 host gene MIR155HG is upregulated, together with miR-155, by TGF-β and can target SOX10, thus controlling miR-155/SOX10 axis and endorsing the expression of mesenchymal markers in LSCC [91].

As regards the role played by tumor infiltrating immune cells, the clinical significance of inflammatory response in peritumoral connective tissue and in neoplastic stroma was evaluated in 181 LSCC patients, using a multiple regression model based on an independent variable represented by infiltrating or expansive types of tumor growth to evaluate the probability of LNM occurrence [201]. In this study, the authors analyzed both plasma cells and eosinophil infiltration. They found an inverse correlation with inflammation and nodal involvement, while no correlation was observed in the case of eosinophil infiltration. However, eosinophil involvement in inflammation and tumor neovascularization in LSCC cannot be excluded, given the release of growth factors (i.e., VEGF) and other proangiogenic mediators [202]. Eosinophils may also act as important players for the immune system in the regulation of tumor growth and cancer survival, since cytokines, chemokines, and growth factors derived from their degranulation in TME may be involved in improving T cell-mediated tumor killing [203]. The immune system can counteract cancer initiation and metastasis progression working at the early stages of tumor development by the recognition and elimination of immunogenic cancer cells carried out by cytotoxic cells such as natural killer (NK) and CD8+ T cells [204]. The involvement of immune surveillance in tumor metastasis has been analyzed in recent years through the investigation of the specific role of different leukocyte populations; as a result, systemic hematological markers and peripheral T cell subsets have become promising prognosticators. A Chinese study analyzed, in a small cohort of LSCC patients, the relevance of dendritic cell infiltration in tumor stroma, positively correlating the infiltration with a reduced probability of cervical metastasis and a longer survival time [205]. On these bases, dendritic cell infiltration was proposed as a feasible prognostic index. On the other hand, the presence of neutrophils in tumor microenvironment has not been systematically investigated, despite some slight evidence indicative of disease progression depending on the tumor site and on TGF-β levels [206,207]. Indeed, there has been extensive research regarding the role of tumor associated macrophages (TAMs), whilst little attention has been paid to tumor associated neutrophils (TANs), which can show either tumor-suppressive (N1) or tumor-promoting (N2) phenotype [208]. N2 are known to undergo the chemo-attraction promoted by angiogenic chemokines in the TME, releasing in turn the content of cytoplasmic granules, i.e., MMPs, ROS, proangiogenic cytokines, and cytokines, suppressing immune response. TAN can also promote B cell maturation and proliferation through the expression of a “proliferation inducing ligand” known as APRIL or tumor necrosis factor superfamily member 13 (TNFSF13, also identified as a proliferation-inducing ligand, APRIL) [208]. The role of APRIL as a diagnostic and prognostic LSCC serum biomarker has been demonstrated by Wang et al. [209], who also showed a positive correlation of APRIL expression with ki-67 and NF-κB p65. Moreover, functional in vitro studies revealed that APRIL-silencing inhibited cell proliferation by blocking G1 phase in HEp-2 cells. Simultaneously with this study, another research group demonstrated the direct targeting of APRIL by miR-383 in hepatocellular carcinoma, providing the first evidence of miR-383 decrease in this neoplasm, and proving its correlation with cancer progression and prognosis [210]. A recent study identified tumor infiltrating immune cells and preoperative neutrophil-to-lymphocyte ratio as potential prognostic factors for the recognition of both increased risk of local recurrence and low rate of PFS in LSCC. However, ROC analysis did not show any correlation with mortality rate [211]. More recently, a retrospective study performed on 229 patients with benign, premalignant, and malignant laryngeal lesions demonstrated the predictive role of neutrophil-lymphocyte ratio for the identification of stage, lymph-node, and distant metastasis, as well as its prognostic role for the determination of OS, DFS, and locoregional recurrence free survival [212]. The controversial role of the immune system in LSCC was also investigated by an in-depth analysis of full blood counts and T cell subset distribution with the aim to establish a correlation with patients’ clinical parameters and survival. Neutrophil–lymphocyte and platelet–lymphocyte ratios were both analyzed by Marchi et al. in LSCC patients, where these parameters were significantly increased. Moreover, the same authors correlated the reduced CD4+/CD8+ and CD3+/CD8+ ratios with recurrent disease and the improved amount of CD3+ and CD4+ with LNM [213]. The last finding was fully in agreement with a previous observation by Drennan et al. and was ascribed to the onset of inflammatory reaction in the presence of LNM and advanced stages of disease [214]. The systemic immunological changes observed during tumor progression also include an increased number of T-regs, a depletion of PD-1 expressing T cells, and an overall reduced T cell function. The observed inverse correlation between T-reg and T cell levels is not surprising since it was demonstrated that T-regs characterized by CD127 low/− phenotype suppress effector T cell proliferation at a higher extent in metastasized LSCC patients, compared with healthy individuals or with patients without nodal involvement. Moreover, peripheral T-regs were influenced by the patient’s tumor stage, indicating a role in tumor progression and the possibility to successfully use immunotherapy for LSCC treatment [214]. The possible role of chemokine receptors CCR6 and CCR7, known to orchestrate T cell migration and metastasis, as well as their ligands CCL20, CCL19, and CCL21, were explored to investigate their possible correlation with clinical-pathological characteristics of LSCC [215]. In detail, it was demonstrated that both chemokine receptors were highly expressed in laryngeal cancer tissues, in LNM and in CD4 + CD25 + Foxp3+ T-regs, also showing a direct involvement in driving cancer cell migration and immune tolerance by recruiting CD4 + CD25 + Foxp3+ T-regs to cancer sites and stimulating LSCC onset, invasion, and metastasis. These results suggested a possible function as molecular biomarkers for assessment and prognosis of LSCC. Notably, CCR6 is positively correlated with miR-20a-5p and miR-489, and negatively associated with miR-29-3p, miR-632, and miR-1276 in LSCC [216], while no evidence of miRNA regulation was reported for CCR7 in laryngeal cancer (Figure 3).

## 6. Concluding Remarks and Future Perspectives

Laryngeal cancer is one of the most common malignant forms belonging to head and neck district neoplasms. The diagnostic process and the proper therapeutic planning are a result of the thorough knowledge of natural history, as well as of clinical and molecular factors underlying this disease. LSCC is characterized by local growth and diffusion in neighboring lymph nodes, although it frequently develops distant metastases. Due to the nonspecific symptoms, LSCC diagnosis often occurs in a late phase, resulting in delayed treatment and worse prognosis. A further crucial concern is the attempt to improve patients’ quality of life trying to preserve laryngeal function. This goal could be more easily achieved through early diagnosis and prompt treatment initiation. Today, the advent of emerging molecular and genetic “omics” technologies has given a powerful shove-ahead in LSCC characterization and monitoring, allowing the identification of novel biomolecules as diagnostic and prognostic markers for clinical management of the neo-plasm. Aberrant gene expression and transforming epigenetic events represent a powerful resource to recognize the mechanisms underlying carcinogenesis and to identify new candidate biomarkers. Within the broad variety of molecular factors endowed with therapeutic and/or diagnostic potential, a central role seems to be played by non-coding RNAs, which have aroused growing interest for their multiple biomedical applications in the new era of personalized medicine. They can orchestrate the regulation of multiple cancer-related pathways, actively participating in tumor spreading and MDR phenotype, as well. A favorable chance to improve LSCC monitoring also derives from the clinical application of liquid biopsy, which is so far under investigation and looks promising based on the multiple pieces of evidence outlined for HNSCC. However, despite the remarkable efforts to develop new effective molecules able to target oncogenic factors, a major challenge is still represented by the development of effective chemotherapy agents for progressed metastatic LSCC. Therefore, new pharmacological approaches targeting the molecular mechanisms underlying the circumvention of anticancer drugs’ cytotoxicity are absolutely required. Moving in this direction, a great chance can come from the use of non-coding RNAs, which are engaged in both the acquisition and the maintenance of resistant phenotypes. Finally, in-depth characterization of LSCC cancer cannot be unaware of a pro-inflammatory microenvironment and tumor-infiltrating immune cells, which have been recently examined for their role in EMT process regulation and for their promising prognostic function.

The high number of potential biomarkers currently available still exhibit a series of limitations, mostly due to the integration process of the massive datasets resulting from omic methodologies, availing of computing platforms and standardized analytic systems. Of note, there is also a compelling need to optimize biomarkers’ validation against accepted clinical and pathological parameters. All these factors clearly indicate that the future direction of LSCC treatment must necessarily be centered on tailored interventions resulting from multidisciplinary and standardized approaches.

## Figures and Tables

**Table 1:** Aberrantly expressed genes in LSCC patients.

Gene	Mutation	Regulation	Biological Role	Clinical-Pathological and Prognostic Implications	Refs.
*TP53*	Disruptive (exons 2 through 11)	Down (wild type isoform)	Tumor suppressor	Decreased overall survival HR (95% CI):1.4 (1.1–1.8) (*p* = 0.009) Primary tumor recurrence (*p* = 0.001)	[31,32,33,34,35,37]
*TP63*	-	Abnormal Expression	Tumor suppressor/ oncogene	Cancer development Tumor recurrence Higher death rate	[36]
*IL1A*	-	Down	Anti-tumor immune response	Primary tumor recurrence (*p* = 0.004)	[37]
*RB1*	-	Down	Tumor suppressor	Primary tumor recurrence (*p* = 0.031)	[37]
*STK1*	-	Up	Tumor suppressor	Primary tumor recurrence (*p* = 0.031)	[37]
*LMNA*	-	Down	Transcriptional signaling	Lymph node metastases	[37]
*RECQL4*	-	Down	DNA replication	Lymph node metastases	[37]
*IGF1R*	-	Down	Receptor	Lymph node metastases	[37]
*N33*	-	Up	Tumor suppressor	Lymph node metastases	[37]
*CDKN2D*	-	Up	Signal transduction	Lymph node metastases	[37]
*Cyclin D1*	-	Up	Oncogene	Lymph node metastases Tumor staging Decreased overall survival	[49]
*P21*	-	Up	Tumor suppressor	-	[42]
*FGF3*	-	Up	Signal transduction	-	[42]
*P16*	-	Up	Cell cycle regulator	-	[42]
*P27*	-	Down	Tumor suppressor	Shorter 5-year overall survival (*p* = 0.045) Advanced tumor size (*p* = 0.0023) Lymph node metastasis (*p* = 0.0016) General metastasis occurrence (*p* = 0.0097) Advanced clinical stage (*p* = 0.0008)	[43,44,45]
*NOTCH1*	-	Up (wild type isoform) Down (mutated isoform)	Oncogene/tumor suppressor	Higher clinical stage incidence of lymph node metastasis Histological grade (*p* < 0.05) Shorter overall survival and disease-free survival *p* < 0.01	[56,58,59]
*NOTCH2*	-	Up	Oncogene	Tumorigenesis Lymph node metastasis	[60]
*EGFR*	Exons Deletions (19-del) Insertions (20-ins), Point Missense Mutations (L858R, T790M, G719X, T790M and L858R, L861Q, G719X and L861Q)	Up	Oncogene	Tumor aggressiveness Lymph node metastasis (*p* = 0.534) Decreased overall survival	[48]
*BCL2*	-	Up	Oncogene	Tumor recurrence Reduced survival Chemo-resistance	[38,41]
*BCL2L12*	-	Up	Tumor suppressor	Increased overall survival	[39]
*OGG1*	Silent and missense mutations	Aberrant expression	DNA structure regulation	Higher cancer risk T3–T4 stage (*p* < 0.04) Laryngeal cancer (*p* < 0.02)	[63]
*E-cadherin*	-	Down	Tumor suppressor	Tumor staging Degree of differentiation Lymph node metastasis Supraglottic localization Poor differentiation	[53]
*FGFR3*	Non-synonymous mutations (S249C)	Aberrant expression	Tumor suppressor/ oncogene	Progressing dysplasia Carcinoma progression	[29]
*PIK3CA*	Non-synonymous mutations (E542K)	Aberrant expression	Oncogene	Progressing dysplasia Carcinoma progression	[29]
*TRKB*	-	Up	Oncogene	-	[67,68]
*NAT1/NAT2*	Polymorphism	Aberrant expression	Oncogene	Increased risk of laryngeal cancer	[62]
*PARK7*	-	Up	Oncogene	Cancer cells proliferation Lymph node metastasis Laryngeal cancer localization Clinical stage	[65]
*β-catenin*	-	Up	Oncogene	Cervical metastasis Later tumor (T) stage Decreased tumor differentiation Reduced overall survival (*p* = 0.002)	[53,54]
*ZEB2*	-	Up	Oncogene	Later tumor (T) stage Decreased tumor differentiation Reduced overall survival (*p* = 0.0003)	[54]
*N.cadherin*	-	Up	Oncogene	Later tumor (T) stage Decreased tumor differentiation Reduced overall survival (*p* = 0.003)	[54]
*CTNNA2*	Point mutations Missense mutations Nonsense mutations	Up (mutated isoform) Down (wild type isoform)	Tumor suppressor	Worse prognosis	[55]
*CTNNA3*	Point mutations Missense mutations Nonsense mutations	Up (mutated isoform) Down (wild type isoform)	Tumor suppressor	Worse prognosis	[55]
*JAK3*	Non-synonymous mutations c.2164G > A	Aberrant expression	Oncogene	Non-progressing dysplasia Carcinoma progression	[29]
*MET*	Non-synonymous mutations c.2962C > T	Aberrant expression	Oncogene	Non-progressing dysplasia Carcinoma progression	[29]
*FWXB7*	Non-synonymous mutations c.1273C > A	Aberrant expression	Oncogene	Non-progressing dysplasia Carcinoma progression	[29]
*PTEN*	Point mutations LOH Epigenetic silencing (abnormal methylation)	Aberrant expression	Tumor suppressor	Shorter overall survival Glottic localization Advanced tumor grade (when downregulated)	[64]
*KRAS*	-	Up	Oncogene	Tumorigenesis Invasion Lymph node metastasis Recurrence Decreased overall survival	[49]

**Table 2:** Aberrantly expressed miRNAs and lncRNAs in metastatic LSCC patients.

Non-Coding RNA	Regulation	Sample Type	Biological Role	Target	Clinic-Pathologic and Prognostic Implications	Ref.
**miRNA**						
miR-141	Down	Tissue	Tumor supressor	HOXC6	TNM stage Differentiation degree Lymph node metastasis (*p* < 0.001)	[72]
miR-138	Down	Tissue	Tumor supressor	ZEB2	Distal metastases of primary LC Poor prognosis (*p* < 0.05)	[73]
miR-145	Down	Tissue	Tumor supressor	MYO5A	T stage Cell differentiation Cervical metastatic state (*p* < 0.05)	[74]
miR-203	Down	Tissue	Tumor supressor	ASAP1	Advanced T stage (*p* < 0.002) Differentiation (*p* < 0.001) Lymph node metastasis (*p* = 0.044) Decreased 5-year overall survival (*p* = 0.002)	[75]
miR-204-5p	Down	Tissue	Tumor supressor	FOXC1	Cervical lymph node (*p* = 0.019) Clinical stage (*p* = 0.005)	[76]
miR-143-3p	Down	Tissue	Tumor supressor	KRAS	T classification Differentiation Lymph node metastasis Clinical stage (*p* < 0.05)	[77]
miR-101	Down	Tissue	Tumor supressor	CDK8	T classification (*p* = 0.015) Lymph node metastasis (*p* = 0.044) Clinical stage (*p* = 0.004)	[78]
miR-744-3p	Up	Tissue	Oncogene	PDCD4	Lymph node metastasis (*p* = 0.007)	[83]
miR-21	Up	Tissue	Oncogene	Ras	T classification (*p* = 0.0001) Differentiation (*p* = 0.004) Lymph node metastasis (*p* = 0.0008) Clinical stage (*p* < 0.001)	[84]
miR-149	Down	Tissue	Tumor supressor	-	T Stage (*p* = 0.022) Lymph node metastasis (*p* = 0.018) Differentiation (*p* = 0.036) Shorter overall survival (median survival of 48 months, 95% CI of ratio 0.2536 to 1.385; *p* = 0.0405)	[79]
miR-618, miR-542-5p, let7b, miR-135a, miR-20b, miR-324-3p, miR-886-5p	Up	Tissue	-	-	Lymph node metastasis	[86]
miR486-3p, miR-328, miR-376a, miR-493	Down	Tissue	-	-	Lymph node metastasis	[86]
miR-129-5p	Up	Tissue	Oncogene	APC	T classification (*p* = 0.04) Lymph node metastasis (*p* = 0.02) Clinical stage (*p* = 0.01)	[85]
miR-34a	Down	Tissue	Tumor supressor	Survivin	Histological Differentiation (*p* < 0.0001) Lymphatic metastasis (*p* = 0.0022) TNM stage (*p* = 0.0111)	[81]
miR-195	Down	Tissue	Tumor supressor	DCUN1D1	Shorter overall survival (*p* = 0.029) T stage (*p* < 0.001) Lymph node metastases (*p* = 0.035) Clinical stage (*p* < 0.001)	[82]
miR-21	Up	Serum	-	-	-	[89]
miR-155	Up	Plasma	Oncogene	-	T stage (*p* = 0.001) Lymph node metastases (*p* = 0.007) Tumor size (*p* = 0.033)	[91]
miR-632	Up	Serum	-	-	T stage (*p* = 0.014) Lymph node metastases (*p* = 0.020) Histological grade (*p* = 0.001)	[92]
miR-449a	Down	Tissue	Oncogene	-	Lymph node metastases (*p* < 0.01) (ROC sensitivity = 0.55, specificity = 0.76, AUC = 0.67)	[87]
miR-130a	Down	Plasma	-		Grade I of cachexia (*p* = 0.044) Cachexia grade I vs. grade II Sensitivity of 63.6% and specificity of 64.7% (AUC = 0.663; *p* < 0.05) Shorter overall survival (*p* = 0.087 HR = 2.582)	[94]
**lncRNA**						
HOXA11-AS	Up	Tissue	Oncogene	-	T stage (*p* = 0.011) Differentiation (*p* = 0.014) Lymph node metastasis (*p* = 0.026) Clinical stage (*p* = 0.001)	[96]
RGMB-AS1	Up	Tissue	Oncogene	miR-22/NLRP3 axis	T stage (*p* < 0.05) Lymph node metastasis (*p* < 0.05) Shorter Overall Survival and Disease Free Survival (*p* < 0.05)	[97]
NKILA	Down	Tissue	Tumor-supressor	NF-kB pathway	T stage (*p* = 0.002) Lymph node metastasis Clinical stage (*p* < 0.001) Shorter overall survival	[98]
H19	Up	Tissue	Oncogene	miR-148a-3p/DNMT1 axis	T stage (*p* < 0.01) Lymph node metastasis (*p* < 0.01) Differentiation (*p* < 0.017) Clinical stage (*p* < 0.01) Shorter overall survival (*p* = 0.003)	[99]
TUG1	Up	Tissue	Oncogene	-	T stage (*p* = 0.025) Lymph node metastasis (*p* = 0.014) Clinical stage (*p* = 0.003)	[100]
MIR155HG	Up	Tissue	Oncogene	miR-155/ SOX10 axis	T stage (*p* < 0.001) Lymph node metastases (*p* < 0.01) Differentiation (*p* < 0.05)	[91]
NEAT1	Up	Tissue	Oncogene	miR-107/CDK6 axis	T stage (*p* < 0.001) Lymph node metastases (*p* < 0.05) Histological grade (*p* < 0.001)	[101]
ST7-AS1	Up	Tissue	Oncogene	CARM1/ SOX2 axis	T stage (*p* < 0.001) Lymph node metastases (*p* < 0.001) Shorter overall survival (*p* = 0.0023)	[102]
XIST	Up	Tissue	Oncogene	miR-124/EZH2 axis	T stage (*p* < 0.05)	[103]
HOTAIR	Up	Serum	-	-	T stage (*p* < 0.0038) Lymph node metastases (*p* = 0.0003) Clinical stage (*p* = 0.0061)	[90]
UCA1	Up	Serum	-	Wnt/β-catenin pathway	Distant metastasis Shorter overall survival (*p* = 0.032)	[104]
snaR	Up	Plasma	Oncogene	TGF-β1	AJCC stages (*p* < 0.05) Shorter overall survival (*p* < 0.05)	[105]

## References

[1] Piotrowski I., Zhu X., Saccon T.D., Ashiqueali S., Schneider A., de Carvalho Nunes A.D., Noureddine S., Sobecka A., Barczak W., Szewczyk M. (2021). MiRNAs as Biomarkers for Diagnosing and Predicting Survival of Head and Neck Squamous Cell Carcinoma Patients. Cancers.

[2] Sung H., Ferlay J., Siegel R.L., Laversanne M., Soerjomataram I., Jemal A., Bray F. (2021). Global Cancer Statistics 2020: GLOBOCAN Estimates of Incidence and Mortality Worldwide for 36 Cancers in 185 Countries. CA Cancer J. Clin..

[3] Cancer of the Larynx—Cancer Stat Facts. https://seer.cancer.gov/statfacts/html/laryn.html.

[4] Kreimer A.R., Clifford G.M., Boyle P., Franceschi S. (2005). Human Papillomavirus Types in Head and Neck Squamous Cell Carcinomas Worldwide: A Systematic Review. Cancer Epidemiol. Prev. Biomark..

[5] Orell-Kotikangas H., Österlund P., Mäkitie O., Saarilahti K., Ravasco P., Schwab U., Mäkitie A.A. (2017). Cachexia at Diagnosis Is Associated with Poor Survival in Head and Neck Cancer Patients. Acta OtoLaryngol..

[6] O’Neill J.P., Shaha A.R. (2011). Nutrition Management of Patients with Malignancies of the Head and Neck. Surg. Clin. N. Am..

[7] Santos M., Monteiro E. (2021). Time between Diagnosis and Treatment of Hypopharynx and Larynx Cancer: Are Longer Delays Associated with Higher Discrepancy between Clinical and Pathological Staging?. Int. Arch. Otorhinolaryngol..

[8] Baird B.J., Sung C.K., Beadle B.M., Divi V. (2018). Treatment of Early-Stage Laryngeal Cancer: A Comparison of Treatment Options. Oral Oncol..

[9] García Lorenzo J., Montoro Martínez V., Rigo Quera A., Codina Aroca A., López Vilas M., Quer Agustí M., León Vintró X. (2017). Modifications in the Treatment of Advanced Laryngeal Cancer throughout the Last 30 Years. Eur. Arch. OtoRhinoLaryngol..

[10] Forastiere A.A., Ismaila N., Lewin J.S., Nathan C.A., Adelstein D.J., Eisbruch A., Fass G., Fisher S.G., Laurie S.A., Le Q.-T. (2018). Use of Larynx-Preservation Strategies in the Treatment of Laryngeal Cancer: American Society of Clinical Oncology Clinical Practice Guideline Update. J. Clin. Oncol..

[11] Singh A., Qayyumi B., Chaturvedi P. (2020). An Update on Surgical Margins in the Head Neck Squamous Cell Carcinoma: Assessment, Clinical Outcome, and Future Directions. Curr. Oncol. Rep..

[12] Beibei Y., Rong Y., Yunfei Y., Wenchao Z. (2021). Research Progress Regarding Surgical Margins, Molecular Margins, and Prognosis of Laryngeal Carcinoma. Ear Nose Throat J..

[13] Holliday E.B., Smith B.D., Gross N.D., Fuller C.D., Rosenthal D.I., The American Cancer Society (2018). Larynx Cancer. The American Cancer Society’s Oncology in Practice.

[14] Nocini R., Molteni G., Mattiuzzi C., Lippi G. (2020). Updates on Larynx Cancer Epidemiology. Chin. J. Cancer Res..

[15] Pezzuto F., Buonaguro L., Caponigro F., Ionna F., Starita N., Annunziata C., Buonaguro F.M., Tornesello M.L. (2015). Update on Head and Neck Cancer: Current Knowledge on Epidemiology, Risk Factors, Molecular Features and Novel Therapies. Oncology.

[16] Chen X., Gao L., Sturgis E.M., Liang Z., Zhu Y., Xia X., Zhu X., Chen X., Li G., Gao Z. (2017). HPV16 DNA and Integration in Normal and Malignant Epithelium: Implications for the Etiology of Laryngeal Squamous Cell Carcinoma. Ann. Oncol..

[17] Lifsics A., Groma V., Cistjakovs M., Skuja S., Deksnis R., Murovska M. (2021). Identification of High-Risk Human Papillomavirus DNA, P16, and E6/E7 Oncoproteins in Laryngeal and Hypopharyngeal Squamous Cell Carcinomas. Viruses.

[18] Thompson L.D.R. (2017). Laryngeal Dysplasia, Squamous Cell Carcinoma, and Variants. Surg. Pathol. Clin..

[19] García J.J., Richardson M.S. (2011). Common Lesions of the Larynx and Hypopharynx. Surg. Pathol. Clin..

[20] Ciolofan M.S., Vlăescu A.N. (2017). Clinical, Histological and Immunohistochemical Evaluation of Larynx Cancer. Curr. Health Sci. J..

[21] Marioni G., Marchese-Ragona R., Cartei G., Marchese F., Staffieri A. (2006). Current Opinion in Diagnosis and Treatment of Laryngeal Carcinoma. Cancer Treat. Rev..

[22] Dispenza F., De Stefano A., Marchese D., Martines F., Dispenza C. (2012). Management of Laryngeal Precancerous Lesions. Auris. Nasus. Larynx.

[23] Johnson D.E., Burtness B., Leemans C.R., Lui V.W.Y., Bauman J.E., Grandis J.R. (2020). Head and Neck Squamous Cell Carcinoma. Nat. Rev. Dis. Primer.

[24] Hu Y., Liu H. (2015). MicroRNA-10a-5p and MicroRNA-34c-5p in Laryngeal Epithelial Premalignant Lesions: Differential Expression and Clinicopathological Correlation. Eur. Arch. OtoRhinoLaryngol..

[25] Tuncturk F.R., Akalin I., Uzun L., Zenginkinet T. (2021). Comparison of MiRNA Expressions among Benign, Premalignant and Malignant Lesions of the Larynx: Could They Be Transformation Biomarkers?. J. Otolaryngol. Head Neck Surg..

[26] Ying X., Kai W., Wei G., Chunming Z., Fuhui H., Shuxin W., Binquan W. (2013). MicroRNA-106b Regulates the Tumor Suppressor RUNX3 in Laryngeal Carcinoma Cells. FEBS Lett..

[27] Daquan W., Tian W., Shen N., Danzheng L., Xinsheng H. (2021). Decrement of Prognostic Nutrition Index in Laryngeal Diseases: From Precancerous Lesion to Squamous Cell Carcinoma. Acta OtoLaryngol..

[28] Lan L., Cao H., Chi W., Meng W., Zhao L., Cui W., Wang B. (2020). Aberrant DNA Hyper-Methylation-Silenced LINC00886 Gene Accelerates Malignant Progression of Laryngeal Carcinoma. Pathol. Res. Pract..

[29] Manterola L., Aguirre P., Larrea E., Arestín M., Gaafar A., Elorriaga K., Goicoechea I., Armesto M., Fernández-Mercado M., Zabalza I. (2018). Mutational Profiling Can Identify Laryngeal Dysplasia at Risk of Progression to Invasive Carcinoma. Sci. Rep..

[30] Zhou G., Liu Z., Myers J.N. (2016). TP53 Mutations in Head and Neck Squamous Cell Carcinoma and Their Impact on Disease Progression and Treatment Response. J. Cell. Biochem..

[31] Poeta M.L., Manola J., Goldwasser M.A., Forastiere A., Benoit N., Califano J.A., Ridge J.A., Goodwin J., Kenady D., Saunders J. (2007). TP53 Mutations and Survival in Squamous-Cell Carcinoma of the Head and Neck. N. Engl. J. Med..

[32] Leemans C.R., Braakhuis B.J.M., Brakenhoff R.H. (2011). The Molecular Biology of Head and Neck Cancer. Nat. Rev. Cancer.

[33] Chrysovergis A., Papanikolaou V., Tsiambas E., Stavraka C., Ragos V., Peschos D., Psyrri A., Mastronikolis N., Kyrodimos E. (2019). P53/MDM2 Co-Expression in Laryngeal Squamous Cell Carcinoma Based on Digital Image Analysis. Anticancer Res..

[34] Osman I., Sherman E., Singh B., Venkatraman E., Zelefsky M., Bosl G., Scher H., Shah J., Shaha A., Kraus D. (2002). Alteration of P53 Pathway in Squamous Cell Carcinoma of the Head and Neck: Impact on Treatment Outcome in Patients Treated with Larynx Preservation Intent. J. Clin. Oncol..

[35] Zhang X., Wang L., Liu S., Ouyang X., Liang C. (2002). The relationship of p53 gene mutation to cell differentiation and metastasis of laryngeal squamous cell carcinoma. Zhonghua Yi Xue Yi Chuan Xue Za Zhi Chin. J. Med. Genet..

[36] Pruneri G., Pignataro L., Manzotti M., Carboni N., Ronchetti D., Neri A., Cesana B.M., Viale G. (2002). P63 in Laryngeal Squamous Cell Carcinoma: Evidence for a Role of TA-P63 down-Regulation in Tumorigenesis and Lack of Prognostic Implications of P63 Immunoreactivity. Lab. Investig. J. Tech. Methods Pathol..

[37] Marcos C.Á., Alonso-Guervós M., Prado N.R., Gimeno T.S., Iglesias F.D., Hermsen M., Llorente J.L. (2011). Genetic Model of Transformation and Neoplastic Progression in Laryngeal Epithelium. Head Neck.

[38] Pruneri G., Pignataro L., Carboni N., Ronchetti D., Cesana B.M., Ottaviani A., Neri A., Buffa R. (1998). Clinical Relevance of P53 and Bcl-2 Protein over-Expression in Laryngeal Squamous-Cell Carcinoma. Int. J. Cancer.

[39] Giotakis A.I., Lazaris A.C., Kataki A., Kontos C.K., Giotakis E.I. (2019). Positive BCL2L12 Expression Predicts Favorable Prognosis in Patients with Laryngeal Squamous Cell Carcinoma. Cancer Biomark..

[40] Chrysovergis A., Papanikolaou V.S., Tsiambas E., Ragos V., Peschos D., Kyrodimos E. (2019). Digital Analysis of BCL2 Expression in Laryngeal Squamous Cell Carcinoma. Anticancer Res..

[41] Giotakis A.I., Kontos C.K., Manolopoulos L.D., Sismanis A., Konstadoulakis M.M., Scorilas A. (2016). High BAX/BCL2 MRNA Ratio Predicts Favorable Prognosis in Laryngeal Squamous Cell Carcinoma, Particularly in Patients with Negative Lymph Nodes at the Time of Diagnosis. Clin. Biochem..

[42] Jovanovic I.P., Radosavljevic G.D., Simovic-Markovic B.J., Stojanovic S.P., Stefanovic S.M., Pejnovic N.N., Arsenijevic N.N. (2014). Clinical Significance of Cyclin D1, FGF3 and P21 Protein Expression in Laryngeal Squamous Cell Carcinoma. J. BUON Off. J. Balk. Union Oncol..

[43] Pruneri G., Pignataro L., Carboni N., Buffa R., Di Finizio D., Cesana B.M., Neri A. (1999). Clinical Relevance of Expression of the CIP/KIP Cell-Cycle Inhibitors P21 and P27 in Laryngeal Cancer. J. Clin. Oncol..

[44] Fan G.K., Fujieda S., Sunaga H., Tsuzuki H., Ito N., Saito H. (1999). Expression of Protein P27 Is Associated with Progression and Prognosis in Laryngeal Cancer. Laryngoscope.

[45] Tamura N., Dong Y., Sui L., Tai Y., Sugimoto K., Nagahata S., Tokuda M. (2001). Cyclin-Dependent Kinase Inhibitor P27 Is Related to Cell Proliferation and Prognosis in Laryngeal Squamous Cell Carcinomas. J. Laryngol. Otol..

[46] Peschos D., Tsanou E., Stefanou D., Damala C., Vougiouklakis T., Mitselou A., Agnantis N.J. (2004). Expression of Cyclin-Dependent Kinases Inhibitors P21(WAF1) and P27(KIP1) in Benign, Premalignant and Malignant Laryngeal Lesions. Correlation with Cell Cycle Regulatory Proteins. Vivo Athens Greece.

[47] Politi A., Tsiambas E., Mastronikolis N.S., Peschos D., Asproudis I., Kyrodimos E., Armata I.E., Chrysovergis A., Asimakopoulos A., Papanikolaou V.S. (2019). Combined EGFR/ALK Expression Analysis in Laryngeal Squamous Cell Carcinoma. Vivo Athens Greece.

[48] Cercelaru L., Stepan A.E., Mărgăritescu C., Osman A., Popa I.-C., Simionescu C.E., Mărgăritescu O. (2017). EGFR Immunoexpression in Laryngeal Squamous Cell Carcinoma. Curr. Health Sci. J..

[49] Lin X., Wen G., Wang S., Lu H., Li C., Wang X. (2019). Expression and Role of EGFR, Cyclin D1 and KRAS in Laryngocarcinoma Tissues. Exp. Ther. Med..

[50] Jung A.R., Jung C.-H., Noh J.K., Lee Y.C., Eun Y.-G. (2020). Epithelial-Mesenchymal Transition Gene Signature Is Associated with Prognosis and Tumor Microenvironment in Head and Neck Squamous Cell Carcinoma. Sci. Rep..

[51] Larizadeh M.H., Damghani M.A., Tabrizchi H., Mirshekari T.R. (2009). Expression of E-Cadherin in Squamous Cell Carcinoma of the Larynx and its Correlation with Clinicopathological Features. J. Med. Sci..

[52] Ahmed R.A., Shawky A.E.-A., Hamed R.H. (2014). Prognostic Significance of Cyclin D1 and E-Cadherin Expression in Laryngeal Squamous Cell Carcinoma. Pathol. Oncol. Res..

[53] Nardi C.E., Dedivitis R.A., de Almeida R.C., de Matos L.L., Cernea C.R. (2018). The Role of E-Cadherin and β-Catenin in Laryngeal Cancer. Oncotarget.

[54] Zhu G.-J., Song P.-P., Zhou H., Shen X.-H., Wang J.-G., Ma X.-F., Gu Y.-J., Liu D.-D., Feng A.-N., Qian X.-Y. (2018). Role of Epithelial-Mesenchymal Transition Markers E-Cadherin, N-Cadherin, β-Catenin and ZEB2 in Laryngeal Squamous Cell Carcinoma. Oncol. Lett..

[55] Fanjul-Fernández M., Quesada V., Cabanillas R., Cadiñanos J., Fontanil T., Obaya A., Ramsay A.J., Llorente J.L., Astudillo A., Cal S. (2013). Cell-Cell Adhesion Genes CTNNA2 and CTNNA3 Are Tumour Suppressors Frequently Mutated in Laryngeal Carcinomas. Nat. Commun..

[56] Sun W., Gaykalova D.A., Ochs M.F., Mambo E., Arnaoutakis D., Liu Y., Loyo M., Agrawal N., Howard J., Li R. (2014). Activation of the NOTCH Pathway in Head and Neck Cancer. Cancer Res..

[57] Fukusumi T., Califano J.A. (2018). The NOTCH Pathway in Head and Neck Squamous Cell Carcinoma. J. Dent. Res..

[58] Li D., Dong P., Wu C., Cao P., Zhou L. (2014). Notch1 Overexpression Associates with Poor Prognosis in Human Laryngeal Squamous Cell Carcinoma. Ann. Otol. Rhinol. Laryngol..

[59] Dai M.-Y., Fang F., Zou Y., Yi X., Ding Y.-J., Chen C., Tao Z.-Z., Chen S.-M. (2015). Downregulation of Notch1 Induces Apoptosis and Inhibits Cell Proliferation and Metastasis in Laryngeal Squamous Cell Carcinoma. Oncol. Rep..

[60] Zou Y., Fang F., Ding Y.-J., Dai M.-Y., Yi X., Chen C., Tao Z.-Z., Chen S.-M. (2016). Notch 2 Signaling Contributes to Cell Growth, Anti-Apoptosis and Metastasis in Laryngeal Squamous Cell Carcinoma. Mol. Med. Rep..

[61] Henning S., Cascorbi I., Münchow B., Jahnke V., Roots I. (1999). Association of Arylamine N-Acetyltransferases NAT1 and NAT2 Genotypes to Laryngeal Cancer Risk. Pharmacogenetics.

[62] Varzim G., Monteiro E., Silva R., Pinheiro C., Lopes C. (2002). Polymorphisms of Arylamine N-Acetyltransferase (NAT1 and NAT2) and Larynx Cancer Susceptibility. J. Oto-Rhino-Laryngol. Its Relat. Spec..

[63] Mahjabeen I., Masood N., Baig R.M., Sabir M., Inayat U., Malik F.A., Kayani M.A. (2012). Novel Mutations of OGG1 Base Excision Repair Pathway Gene in Laryngeal Cancer Patients. Fam. Cancer.

[64] Mastronikolis N.S., Tsiambas E., Papadas T.A., Karameris A., Ragos V., Peschos D., Mastronikolis S.N., Papadas A.T., Liatsos C., Armata I.E. (2017). Deregulation of PTEN Expression in Laryngeal Squamous Cell Carcinoma Based on Tissue Microarray Digital Analysis. Anticancer Res..

[65] Zhu X.-L., Wang Z.-F., Lei W.-B., Zhuang H.-W., Hou W.-J., Wen Y.-H., Wen W.-P. (2012). Tumorigenesis Role and Clinical Significance of DJ-1, a Negative Regulator of PTEN, in Supraglottic Squamous Cell Carcinoma. J. Exp. Clin. Cancer Res..

[66] Kim R.H., Peters M., Jang Y., Shi W., Pintilie M., Fletcher G.C., DeLuca C., Liepa J., Zhou L., Snow B. (2005). DJ-1, a Novel Regulator of the Tumor Suppressor PTEN. Cancer Cell.

[67] Jiang L., Wang Z., Liu C., Gong Z., Yang Y., Kang H., Li Y., Hu G. (2017). TrkB Promotes Laryngeal Cancer Metastasis via Activation PI3K/AKT Pathway. Oncotarget.

[68] Yilmaz T., Jiffar T., de la Garza G., Lin H., Milas Z., Takahashi Y., Hanna E., MacIntyre T., Brown J.L., Myers J.N. (2010). Theraputic Targeting of Trk Supresses Tumor Proliferation and Enhances Cisplatin Activity in HNSCC. Cancer Biol. Ther..

[69] Reddy K.B. (2015). MicroRNA (MiRNA) in Cancer. Cancer Cell Int..

[70] Yu X., Li Z. (2015). The Role of MicroRNAs Expression in Laryngeal Cancer. Oncotarget.

[71] Bouyssou J.M.C., Manier S., Huynh D., Issa S., Roccaro A.M., Ghobrial I.M. (2014). Regulation of MicroRNAs in Cancer Metastasis. Biochim. Biophys. Acta.

[72] Chen L., Sun D.-Z., Fu Y.-G., Yang P.-Z., Lv H.-Q., Gao Y., Zhang X.-Y. (2020). Upregulation of MicroRNA-141 Suppresses Epithelial-Mesenchymal Transition and Lymph Node Metastasis in Laryngeal Cancer through HOXC6-Dependent TGF-β Signaling Pathway. Cell. Signal..

[73] Gao S., Wang J., Xie J., Zhang T., Dong P. (2015). Role of MiR-138 in the Regulation of Larynx Carcinoma Cell Metastases. Tumor Biology.

[74] Zhao X., Zhang W., Ji W. (2018). MYO5A Inhibition by MiR-145 Acts as a Predictive Marker of Occult Neck Lymph Node Metastasis in Human Laryngeal Squamous Cell Carcinoma. OncoTargets Ther..

[75] Tian L., Li M., Ge J., Guo Y., Sun Y., Liu M., Xiao H. (2014). MiR-203 Is Downregulated in Laryngeal Squamous Cell Carcinoma and Can Suppress Proliferation and Induce Apoptosis of Tumours. Tumour Biol..

[76] Gao W., Wu Y., He X., Zhang C., Zhu M., Chen B., Liu Q., Qu X., Li W., Wen S. (2017). MicroRNA-204-5p Inhibits Invasion and Metastasis of Laryngeal Squamous Cell Carcinoma by Suppressing Forkhead Box C1. J. Cancer.

[77] Zhang F., Cao H. (2019). MicroRNA-143-3p Suppresses Cell Growth and Invasion in Laryngeal Squamous Cell Carcinoma via Targeting the K-Ras/Raf/MEK/ERK Signaling Pathway. Int. J. Oncol..

[78] Li M., Tian L., Ren H., Chen X., Wang Y., Ge J., Wu S., Sun Y., Liu M., Xiao H. (2015). MicroRNA-101 Is a Potential Prognostic Indicator of Laryngeal Squamous Cell Carcinoma and Modulates CDK8. J. Transl. Med..

[79] Xu Y., Lin Y.-P., Yang D., Zhang G., Zhou H.-F. (2016). Clinical Significance of MiR-149 in the Survival of Patients with Laryngeal Squamous Cell Carcinoma. BioMed Res. Int..

[80] Misso G., Di Martino M.T., De Rosa G., Farooqi A.A., Lombardi A., Campani V., Zarone M.R., Gullà A., Tagliaferri P., Tassone P. (2014). Mir-34: A New Weapon against Cancer?. Mol. Ther. Nucleic Acids.

[81] Shen Z., Zhan G., Ye D., Ren Y., Cheng L., Wu Z., Guo J. (2012). MicroRNA-34a Affects the Occurrence of Laryngeal Squamous Cell Carcinoma by Targeting the Antiapoptotic Gene Survivin. Med. Oncol. Northwood Lond. Engl..

[82] Shuang Y., Li C., Zhou X., Huang Y., Zhang L. (2017). MicroRNA-195 Inhibits Growth and Inva-sion of Laryngeal Carcinoma Cells by Directly Targeting DCUN1D1. Oncol. Rep..

[83] Li J.Z.-H., Gao W., Lei W.-B., Zhao J., Chan J.Y.-W., Wei W.I., Ho W.-K., Wong T.-S. (2016). MicroRNA 744-3p Promotes MMP-9-Mediated Metastasis by Simultaneously Suppressing PDCD4 and PTEN in Laryngeal Squamous Cell Carcinoma. Oncotarget.

[84] Ren J., Zhu D., Liu M., Sun Y., Tian L. (2010). Downregulation of MiR-21 Modulates Ras Expression to Promote Apoptosis and Suppress Invasion of Laryngeal Squamous Cell Carcinoma. Eur. J. Cancer.

[85] Li M., Tian L., Wang L., Yao H., Zhang J., Lu J., Sun Y., Gao X., Xiao H., Liu M. (2013). Down-Regulation of MiR-129-5p Inhibits Growth and Induces Apoptosis in Laryngeal Squamous Cell Carcinoma by Targeting APC. PLoS ONE.

[86] Ricciardiello F., Capasso R., Kawasaki H., Abate T., Oliva F., Lombardi A., Misso G., Ingrosso D., Leone C.A., Iengo M. (2017). A MiRNA Signature Suggestive of Nodal Metastases from Laryngeal Carcinoma. Acta Otorhinolaryngol. Ital..

[87] Kawasaki H., Takeuchi T., Ricciardiello F., Lombardi A., Biganzoli E., Fornili M., De Bortoli D., Mesolella M., Cossu A.M., Scrima M. (2020). Definition of MiRNA Signatures of Nodal Metastasis in LCa: MiR-449a Targets Notch Genes and Suppresses Cell Migration and Invasion. Mol. Ther. Nucleic Acids.

[88] Zhang J., Li S., Li L., Li M., Guo C., Yao J., Mi S. (2015). Exosome and Exosomal MicroRNA: Trafficking, Sorting, and Function. Genom. Proteom. Bioinform..

[89] Cheng G. (2015). Circulating MiRNAs: Roles in Cancer Diagnosis, Prognosis and Therapy. Adv. Drug Deliv. Rev..

[90] Wang J., Zhou Y., Lu J., Sun Y., Xiao H., Liu M., Tian L. (2014). Combined Detection of Serum Exosomal MiR-21 and HOTAIR as Diagnostic and Prognostic Biomarkers for Laryngeal Squamous Cell Carcinoma. Med. Oncol. Northwood Lond. Engl..

[91] Wang J.L., Wang X., Yang D., Shi W.J. (2016). The Expression of MicroRNA-155 in Plasma and Tissue Is Matched in Human Laryngeal Squamous Cell Carcinoma. Yonsei Med. J..

[92] Cao Y.-C., Song L.-Q., Xu W.-W., Qi J.-J., Wang X.-Y., Su Y. (2020). Serum MiR-632 Is a Potential Marker for the Diagnosis and Prognosis in Laryngeal Squamous Cell Carcinoma. Acta Oto-laryngol..

[93] Cao Z., Zhao K., Jose I., Hoogenraad N.J., Osellame L.D. (2021). Biomarkers for Cancer Cachexia: A Mini Review. Int. J. Mol. Sci..

[94] Powrózek T., Mlak R., Brzozowska A., Mazurek M., Gołębiowski P., Małecka-Massalska T. (2018). MiRNA-130a Significantly Improves Accuracy of SGA Nutritional Assessment Tool in Prediction of Malnutrition and Cachexia in Radiotherapy-Treated Head and Neck Cancer Patients. Cancers.

[95] Bhan A., Soleimani M., Mandal S.S. (2017). Long Noncoding RNA and Cancer: A New Paradigm. Cancer Res..

[96] Qu L., Jin M., Yang L., Sun C., Wang P., Li Y., Tian L., Liu M., Sun Y. (2018). Expression of Long Non-Coding RNA HOXA11-AS Is Correlated with Progression of Laryngeal Squamous Cell Carcinoma. Am. J. Transl. Res..

[97] Xu Z., Xi K. (2019). LncRNA RGMB-AS1 Promotes Laryngeal Squamous Cell Carcinoma Cells Progression via Sponging MiR-22/NLRP3 Axis. Biomed. Pharmacother. Biomed. Pharmacother..

[98] Yang T., Li S., Liu J., Yin D., Yang X., Tang Q. (2018). LncRNA-NKILA/NF-ΚB Feedback Loop Modulates Laryngeal Cancer Cell Proliferation, Invasion, and Radioresistance. Cancer Med..

[99] Wu T., Qu L., He G., Tian L., Li L., Zhou H., Jin Q., Ren J., Wang Y., Wang J. (2016). Regulation of Laryngeal Squamous Cell Cancer Progression by the LncRNA H19/MiR-148a-3p/DNMT1 Axis. Oncotarget.

[100] Zhang Z., Wang X., Cao S., Han X., Wang Z., Zhao X., Liu X., Li G., Pan X., Lei D. (2018). The Long Noncoding RNA TUG1 Promotes Laryngeal Cancer Proliferation and Migration. Cell. Physiol. Biochem. Cell. Physiol. Biochem..

[101] Wang P., Wu T., Zhou H., Jin Q., He G., Yu H., Xuan L., Wang X., Tian L., Sun Y. (2016). Long Noncoding RNA NEAT1 Promotes Laryngeal Squamous Cell Cancer through Regulating MiR-107/CDK6 Pathway. J. Exp. Clin. Cancer Res..

[102] Qin H., Xu J., Gong L., Jiang B., Zhao W. (2019). The Long Noncoding RNA ST7-AS1 Promotes Laryngeal Squamous Cell Carcinoma by Stabilizing CARM1. Biochem. Biophys. Res. Commun..

[103] Xiao D., Cui X., Wang X. (2019). Long Noncoding RNA XIST Increases the Aggressiveness of Laryngeal Squamous Cell Carcinoma by Regulating MiR-124-3p/EZH2. Exp. Cell Res..

[104] Sun S., Gong C., Yuan K. (2019). LncRNA UCA1 Promotes Cell Proliferation, Invasion and Migration of Laryngeal Squamous Cell Carcinoma Cells by Activating Wnt/β-Catenin Signaling Pathway. Exp. Ther. Med..

[105] Liang K., Yang Y., Zha D., Yue B., Qiu J., Zhang C. (2018). Overexpression of LncRNA SnaR Is Correlated with Progression and Predicts Poor Survival of Laryngeal Squamous Cell Carcinoma. J. Cell. Biochem..

[106] Mäbert K., Cojoc M., Peitzsch C., Kurth I., Souchelnytskyi S., Dubrovska A. (2014). Cancer Bi-omarker Discovery: Current Status and Future Perspectives. Int. J. Radiat. Biol..

[107] Yap T.A., Lorente D., Omlin A., Olmos D., de Bono J.S. (2014). Circulating Tumor Cells: A Multi-Functional Biomarker. Clin. Cancer Res. Off. J. Am. Assoc. Cancer Res..

[108] Yong E. (2014). Cancer Biomarkers: Written in Blood. Nature.

[109] Chan K.C.A., Jiang P., Zheng Y.W.L., Liao G.J.W., Sun H., Wong J., Siu S.S.N., Chan W.C., Chan S.L., Chan A.T.C. (2013). Cancer Genome Scanning in Plasma: Detection of Tumor-Associated Copy Number Aberrations, Single-Nucleotide Variants, and Tumoral Heterogeneity by Massively Parallel Sequencing. Clin. Chem..

[110] Balgkouranidou I., Chimonidou M., Milaki G., Tsarouxa E.G., Kakolyris S., Welch D.R., Georgoulias V., Lianidou E.S. (2014). Breast Cancer Metastasis Suppressor-1 Promoter Methylation in Cell-Free DNA Provides Prognostic Information in Non-Small Cell Lung Cancer. Br. J. Cancer.

[111] Sanchez-Cespedes M., Esteller M., Wu L., Nawroz-Danish H., Yoo G.H., Koch W.M., Jen J., Herman J.G., Sidransky D. (2000). Gene Promoter Hypermethylation in Tumors and Serum of Head and Neck Cancer Patients. Cancer Res..

[112] Schröck A., Leisse A., de Vos L., Gevensleben H., Dröge F., Franzen A., Wachendörfer M., Schröck F., Ellinger J., Teschke M. (2017). Free-Circulating Methylated DNA in Blood for Diagnosis, Staging, Prognosis, and Monitoring of Head and Neck Squamous Cell Carcinoma Patients: An Observational Prospective Cohort Study. Clin. Chem..

[113] Chan K.C.A., Hung E.C.W., Woo J.K.S., Chan P.K.S., Leung S.-F., Lai F.P.T., Cheng A.S.M., Yeung S.W., Chan Y.W., Tsui T.K.C. (2013). Early Detection of Nasopharyngeal Carcinoma by Plasma Epstein-Barr Virus DNA Analysis in a Surveillance Program. Cancer.

[114] Campitelli M., Jeannot E., Peter M., Lappartient E., Saada S., de la Rochefordière A., Fourchotte V., Alran S., Petrow P., Cottu P. (2012). Human Papillomavirus Mutational Insertion: Specific Marker of Circulating Tumor DNA in Cervical Cancer Patients. PLoS ONE.

[115] Stroun M., Lyautey J., Lederrey C., Olson-Sand A., Anker P. (2001). About the Possible Origin and Mechanism of Circulating DNA Apoptosis and Active DNA Release. Clin. Chim. Acta Int. J. Clin. Chem..

[116] van der Vaart M., Pretorius P.J. (2007). The Origin of Circulating Free DNA. Clin. Chem..

[117] Jahr S., Hentze H., Englisch S., Hardt D., Fackelmayer F.O., Hesch R.D., Knippers R. (2001). DNA Fragments in the Blood Plasma of Cancer Patients: Quantitations and Evidence for Their Origin from Apoptotic and Necrotic Cells. Cancer Res..

[118] Bettegowda C., Sausen M., Leary R.J., Kinde I., Wang Y., Agrawal N., Bartlett B.R., Wang H., Luber B., Alani R.M. (2014). Detection of Circulating Tumor DNA in Early- and Late-Stage Human Malignancies. Sci. Transl. Med..

[119] Payne K., Spruce R., Beggs A., Sharma N., Kong A., Martin T., Parmar S., Praveen P., Nankivell P., Mehanna H. (2018). Circulating Tumor DNA as a Biomarker and Liquid Biopsy in Head and Neck Squamous Cell Carcinoma. Head Neck.

[120] Terry S., Savagner P., Ortiz-Cuaran S., Mahjoubi L., Saintigny P., Thiery J.-P., Chouaib S. (2017). New Insights into the Role of EMT in Tumor Immune Escape. Mol. Oncol..

[121] Mazurek A.M., Rutkowski T., Fiszer-Kierzkowska A., Małusecka E., Składowski K. (2016). Assessment of the Total CfDNA and HPV16/18 Detection in Plasma Samples of Head and Neck Squamous Cell Carcinoma Patients. Oral Oncol..

[122] Khandelwal A.R., Greer A.H., Hamiter M., Fermin J.M., McMullen T., Moore-Medlin T., Mills G., Flores J.M., Yin H., Nathan C.-A.O. (2020). Comparing Cell-Free Circulating Tumor DNA Mutational Profiles of Disease-Free and Nonresponders Patients with Oropharyngeal Squamous Cell Carcinoma. Laryngoscope Investig. Otolaryngol..

[123] Wang Y., Springer S., Mulvey C.L., Silliman N., Schaefer J., Sausen M., James N., Rettig E.M., Guo T., Pickering C.R. (2015). Detection of Somatic Mutations and HPV in the Saliva and Plasma of Patients with Head and Neck Squamous Cell Carcinomas. Sci. Transl. Med..

[124] McMullen H., Greer A., Khandelwal A.R., Mickie H., Ma X., Moore-Medlin T., Hong Y., Mills G., Nathan C.-A.O. (2017). Abstract 06: Comparing Mutational Profiles between Cell-Free Circu-lating Tumor DNA and Tumor DNA in Laryngeal Carcinoma Patients. Clin. Cancer Res..

[125] Kawada T., Takahashi H., Sakakura K., Ida S., Mito I., Toyoda M., Chikamatsu K. (2017). Circulating Tumor Cells in Patients with Head and Neck Squamous Cell Carcinoma: Feasibility of Detection and Quantitation. Head Neck.

[126] Nichols A.C., Lowes L.E., Szeto C.C.T., Basmaji J., Dhaliwal S., Chapeskie C., Todorovic B., Read N., Venkatesan V., Hammond A. (2012). Detection of Circulating Tumor Cells in Advanced Head and Neck Cancer Using the CellSearch System. Head Neck.

[127] Rizzo M.I., Ralli M., Nicolazzo C., Gradilone A., Carletti R., Di Gioia C., De Vincentiis M., Greco A. (2020). Detection of Circulating Tumor Cells in Patients with Laryngeal Cancer Using ScreenCell: Comparative Pre- and Post-Operative Analysis and Association with Prognosis. Oncol. Lett..

[128] Kanwal R., Gupta S. (2012). Epigenetic Modifications in Cancer. Clin. Genet..

[129] Zhou C., Ye M., Ni S., Li Q., Ye D., Li J., Shen Z., Deng H. (2018). DNA Methylation Biomarkers for Head and Neck Squamous Cell Carcinoma. Epigenetics.

[130] Chen Z., Riggs A.D. (2011). DNA Methylation and Demethylation in Mammals. J. Biol. Chem..

[131] Witte T., Plass C., Gerhauser C. (2014). Pan-Cancer Patterns of DNA Methylation. Genome Med..

[132] Rosas S.L., Koch W., da Costa Carvalho M.G., Wu L., Califano J., Westra W., Jen J., Sidransky D. (2001). Promoter Hypermethylation Patterns of P16, O6-Methylguanine-DNA-Methyltransferase, and Death-Associated Protein Kinase in Tumors and Saliva of Head and Neck Cancer Patients. Cancer Res..

[133] Wong T.-S., Gao W., Li Z.-H., Chan J.Y.-W., Ho W.-K. (2012). Epigenetic Dysregulation in Laryngeal Squamous Cell Carcinoma. J. Oncol..

[134] Smigiel R., Sasiadek M., Krecicki T., Ramsey D., Jagielski J., Blin N. (2004). Inactivation of the Cyclin-Dependent Kinase Inhibitor 2A (CDKN2A) Gene in Squamous Cell Carcinoma of the Larynx. Mol. Carcinog..

[135] Padhi S.S., Roy S., Kar M., Saha A., Roy S., Adhya A., Baisakh M., Banerjee B. (2017). Role of CDKN2A/P16 Expression in the Prognostication of Oral Squamous Cell Carcinoma. Oral Oncol..

[136] Todorova T.A., Jordanov S.H., Stancheva G.S., Chalakov I.J., Melnicharov M.B., Kunev K.V., Mitev V.I., Kaneva R.P., Goranova T.E. (2015). Mutational Status of CDKN2A and TP53 Genes in Laryngeal Squamous Cell Carcinoma. Pathol. Oncol. Res..

[137] Pietruszewska W., Rieske P., Murlewska A., Kobos J., Gryczyński M. (2004). Expression of p16 gene and protein in the evaluation of dynamics of laryngeal cancer growth. Otolaryngol. Pol. Pol. Otolaryngol..

[138] Temam S., Bénard J., Dugas C., Trassard M., Gormally E., Soria J.-C., Faivre S., Luboinski B., Marandas P., Hainaut P. (2005). Molecular Detection of Early-Stage Laryngopharyn-geal Squamous Cell Carcinomas. Clin. Cancer Res..

[139] Giefing M., Zemke N., Brauze D., Kostrzewska-Poczekaj M., Luczak M., Szaumkessel M., Pelinska K., Kiwerska K., Tönnies H., Grenman R. (2011). High Resolution ArrayCGH and Expression Profiling Identifies PTPRD and PCDH17/PCH68 as Tumor Suppressor Gene Candidates in Laryngeal Squamous Cell Carcinoma. Genes Chromosomes Cancer.

[140] Byzia E., Soloch N., Bodnar M., Szaumkessel M., Kiwerska K., Kostrzewska-Poczekaj M., Jarmuz-Szymczak M., Szylberg L., Wierzbicka M., Bartochowska A. (2018). Recurrent Transcriptional Loss of the PCDH17 Tumor Suppressor in Laryngeal Squamous Cell Carcinoma Is Partially Mediated by Aberrant Promoter DNA Methylation. Mol. Carcinog..

[141] Shen Z., Lin L., Cao B., Zhou C., Hao W., Ye D. (2018). LZTS2 Promoter Hypermethylation: A Potential Biomarker for the Diagnosis and Prognosis of Laryngeal Squamous Cell Carcinoma. World J. Surg. Oncol..

[142] Starska K., Forma E., Lewy-Trenda I., Papiez P., Wos J., Brys M. (2013). Diagnostic impact of promoter methylation and E-cadherin gene and protein expression levels in laryngeal carcinoma. Contemp. Oncol..

[143] Calmon M.F., Colombo J., Carvalho F., Souza F.P., Filho J.F.G., Fukuyama E.E., Camargo A.A., Caballero O.L.S., Tajara E.H., Cordeiro J.A. (2007). Methylation Profile of Genes CDKN2A (P14 and P16), DAPK1, CDH1, and ADAM23 in Head and Neck Cancer. Cancer Genet. Cytogenet..

[144] Bouras E., Karakioulaki M., Bougioukas K.I., Aivaliotis M., Tzimagiorgis G., Chourdakis M. (2019). Gene Promoter Methylation and Cancer: An Umbrella Review. Gene.

[145] Paluszczak J., Misiak P., Wierzbicka M., Woźniak A., Baer-Dubowska W. (2011). Frequent Hy-permethylation of DAPK, RARbeta, MGMT, RASSF1A and FHIT in Laryngeal Squamous Cell Carcinomas and Adjacent Normal Mucosa. Oral Oncol..

[146] Pierini S., Jordanov S.H., Mitkova A.V., Chalakov I.J., Melnicharov M.B., Kunev K.V., Mitev V.I., Kaneva R.P., Goranova T.E. (2014). Promoter Hypermethylation of CDKN2A, MGMT, MLH1, and DAPK Genes in Laryngeal Squamous Cell Carcinoma and Their Associations with Clinical Profiles of the Patients. Head Neck.

[147] Dikshit R.P., Gillio-Tos A., Brennan P., De Marco L., Fiano V., Martinez-Peñuela J.M., Boffetta P., Merletti F. (2007). Hypermethylation, Risk Factors, Clinical Characteristics, and Survival in 235 Patients with Laryngeal and Hypopharyngeal Cancers. Cancer.

[148] Xu S., Li Y., Lu Y., Huang J., Ren J., Zhang S., Yin Z., Huang K., Wu G., Yang K. (2018). LZTS2 Inhibits PI3K/AKT Activation and Radioresistance in Nasopharyngeal Carcinoma by Interacting with P85. Cancer Lett..

[149] Azarschab P., Stembalska A., Loncar M.B., Pfister M., Sasiadek M.M., Blin N. (2003). Epigenetic Control of E-Cadherin (CDH1) by CpG Methylation in Metastasising Laryngeal Cancer. Oncol. Rep..

[150] Qian X., Ma X., Zhou H., Yu C., Zhang Y., Yang X., Shen X., Gao X. (2016). Expression and Prognostic Value of E-Cadherin in Laryngeal Cancer. Acta OtoLaryngol..

[151] Paksoy M., Hardal U., Caglar C. (2011). Expression of Cathepsin D and E-Cadherin in Primary Laryngeal Cancers Correlation with Neck Lymph Node Involvement. J. Cancer Res. Clin. Oncol..

[152] Rodrigo J.P., Domínguez F., Alvarez C., Manrique C., Herrero A., Suárez C. (2002). Expression of E-Cadherin in Squamous Cell Carcinomas of the Supraglottic Larynx with Correlations to Clinicopathological Features. Eur J Cancer..

[153] Galera-Ruiz H., Ríos-Moreno M.J., González-Cámpora R., Ortega I., Fernández A., García-Escudero A., Galera-Davidson H. (2012). The Cadherin-Catenin Complex in Laryngeal Squamous Cell Carcinoma. Eur. Arch. OtoRhinoLaryngol..

[154] Eriksen J.G., Buffa F.M., Alsner J., Steiniche T., Bentzen S.M., Overgaard J. (2004). Molecular Profiles as Predictive Marker for the Effect of Overall Treatment Time of Radiotherapy in Supraglottic Larynx Squamous Cell Carcinomas. Radiother. Oncol. J. Eur. Soc. Ther. Radiol. Oncol..

[155] Farag A.K., Roh E.J. (2019). Death-Associated Protein Kinase (DAPK) Family Modulators: Current and Future Therapeutic Outcomes. Med. Res. Rev..

[156] Onerci Celebi O., Tezel G.G., Hosal A.S., Cengiz M., Gullu I.H., Hayran M. (2016). Detection of O6-Methylguanine-DNA Methyltransferase Gene Promoter Region Methylation Pattern Using Pyrosequencing and the Effect of Methylation Pattern on Survival, Recurrence, and Chemotherapy Sensitivity in Patients with Laryngeal Cancer. Pathol. Res. Pract..

[157] Paluszczak J., Hemmerling D., Kostrzewska-Poczekaj M., Jarmuż-Szymczak M., Grenman R., Wierzbicka M., Baer-Dubowska W. (2014). Frequent Hypermethylation of WNT Pathway Genes in Laryngeal Squamous Cell Carcinomas. J. Oral Pathol. Med..

[158] Langevin S.M., Stone R.A., Bunker C.H., Lyons-Weiler M.A., LaFramboise W.A., Kelly L., Seethala R.R., Grandis J.R., Sobol R.W., Taioli E. (2011). MicroRNA-137 Promoter Methylation Is Associated with Poorer Overall Survival in Patients with Squamous Cell Carcinoma of the Head and Neck. Cancer.

[159] Shen Z., Zhou C., Li J., Ye D., Li Q., Wang J., Cui X., Chen X., Bao T., Duan S. (2016). Promoter Hypermethylation of MiR-34a Contributes to the Risk, Progression, Metastasis and Poor Survival of Laryngeal Squamous Cell Carcinoma. Gene.

[160] Gaudet F., Hodgson J.G., Eden A., Jackson-Grusby L., Dausman J., Gray J.W., Leonhardt H., Jaenisch R. (2003). Induction of Tumors in Mice by Genomic Hypomethylation. Science.

[161] Liu J., Xu Z.-M., Qiu G.-B., Zheng Z.-H., Sun K.-L., Fu W.-N. (2014). S100A4 Is Upregulated via the Binding of C-Myb in Methylation-Free Laryngeal Cancer Cells. Oncol. Rep..

[162] Liu J., Guo Y., Fu S., Yang M., Sun K.-L., Fu W.-N. (2010). Hypomethylation-Induced Expression of S100A4 Increases the Invasiveness of Laryngeal Squamous Cell Carcinoma. Oncol. Rep..

[163] Housman G., Byler S., Heerboth S., Lapinska K., Longacre M., Snyder N., Sarkar S. (2014). Drug Resistance in Cancer: An Overview. Cancers.

[164] Mansoori B., Mohammadi A., Davudian S., Shirjang S., Baradaran B. (2017). The Different Mechanisms of Cancer Drug Resistance: A Brief Review. Adv. Pharm. Bull..

[165] Zhou J., Zhang J., Ma W., Zhang W., Ke Z., Ma L. (2018). Anti-Tumor Effect of HOTAIR–MiR-613-SNAI2 Axis through Suppressing EMT and Drug Resistance in Laryngeal Squamous Cell Carcinoma. RSC Adv..

[166] Yuan Z., Xiu C., Liu D., Zhou G., Yang H., Pei R., Ding C., Cui X., Sun J., Song K. (2019). Long Noncoding RNA LINC-PINT Regulates Laryngeal Carcinoma Cell Stemness and Chemoresistance through MiR-425-5p/PTCH1/SHH Axis. J. Cell. Physiol..

[167] Li R., Chen S., Zhan J., Li X., Liu W., Sheng X., Lu Z., Zhong R., Chen L., Luo X. (2020). Long Noncoding RNA FOXD2-AS1 Enhances Chemotherapeutic Resistance of Laryngeal Squamous Cell Carcinoma via STAT3 Activation. Cell Death Dis..

[168] Yuan Z., Xiu C., Song K., Pei R., Miao S., Mao X., Sun J., Jia S. (2018). Long Non-Coding RNA AFAP1-AS1/MiR-320a/RBPJ Axis Regulates Laryngeal Carcinoma Cell Stemness and Chemoresistance. J. Cell. Mol. Med..

[169] Jiang Q., Liu S., Hou L., Guan Y., Yang S., Luo Z. (2020). The Implication of LncRNA MALAT1 in Promoting Chemo-Resistance of Laryngeal Squamous Cell Carcinoma Cells. J. Clin. Lab. Anal..

[170] Liu J., Tang Q., Li S., Yang X. (2016). Inhibition of HAX-1 by MiR-125a Reverses Cisplatin Resistance in Laryngeal Cancer Stem Cells. Oncotarget.

[171] Tian L., Zhang J., Ren X., Liu X., Gao W., Zhang C., Sun Y., Liu M. (2017). Overexpression of MiR-26b Decreases the Cisplatin-Resistance in Laryngeal Cancer by Targeting ATF2. Oncotarget.

[172] Wang X., Zhu W., Zhao X., Wang P. (2016). MiR-133a Enhances the Sensitivity of Hep-2 Cells and Vincristine-Resistant Hep-2v Cells to Cisplatin by Downregulating ATP7B Expression. Int. J. Mol. Med..

[173] Lin X.-J., Liu H., Li P., Wang H.-F., Yang A.-K., Di J.-M., Jiang Q.-W., Yang Y., Huang J.-R., Yuan M.-L. (2020). MiR-936 Suppresses Cell Proliferation, Invasion, and Drug Resistance of Laryngeal Squamous Cell Carcinoma and Targets GPR78. Front. Oncol..

[174] Fu Q., Liu P., Sun X., Huang S., Han F., Zhang L., Xu Y., Liu T. (2017). Ribonucleic Acid Inter-ference Knockdown of IL-6 Enhances the Efficacy of Cisplatin in Laryngeal Cancer Stem Cells by down-Regulating the IL-6/STAT3/HIF1 Pathway. Cancer Cell Int..

[175] Sheng X., Li Y., Li Y., Liu W., Lu Z., Zhan J., Xu M., Chen L., Luo X., Cai G. (2019). PLOD2 Contributes to Drug Resistance in Laryngeal Cancer by Promoting Cancer Stem Cell-like Characteristics. BMC Cancer.

[176] Zhu M., Yin F., Yang L., Chen S., Chen R., Zhou X., Jing W., Fan X., Jia R., Wang H. (2014). Contribution of TIP30 to Chemoresistance in Laryngeal Carcinoma. Cell Death Dis..

[177] Li G., Hu X., Sun L., Li X., Li J., Li T., Zhang X. (2018). C-Fos Upregulates P-Glycoprotein, Contributing to the Development of Multidrug Resistance in HEp-2 Laryngeal Cancer Cells with VCR-Induced Resistance. Cell. Mol. Biol. Lett..

[178] Wang L., Sun J., Gao P., Su K., Wu H., Li J., Lou W. (2019). Wnt1-Inducible Signaling Protein 1 Regulates Laryngeal Squamous Cell Carcinoma Glycolysis and Chemoresistance via the YAP1/TEAD1/GLUT1 Pathway. J. Cell. Physiol..

[179] Xu D., Li D.W., Xie J., Chen X.W. (2019). Effect and Mechanism of Survivin on Hypoxia-Induced Multidrug Resistance of Human Laryngeal Carcinoma Cells. BioMed Res. Int..

[180] Coussens L.M., Werb Z. (2002). Inflammation and Cancer. Nature.

[181] Dvorak H.F. (1986). Tumors: Wounds That Do Not Heal. Similarities between Tumor Stroma Generation and Wound Healing. N. Engl. J. Med..

[182] Xu L., Shen B., Chen T., Dong P. (2016). MiR-203 Is Involved in the Laryngeal Carcinoma Pathogenesis via Targeting VEGFA and Cox-2. OncoTargets Ther..

[183] Kyzas P.A., Stefanou D., Agnantis N.J. (2005). COX-2 Expression Correlates with VEGF-C and Lymph Node Metastases in Patients with Head and Neck Squamous Cell Carcinoma. Mod. Pathol..

[184] Morita Y., Hata K., Nakanishi M., Nishisho T., Yura Y., Yoneda T. (2012). Cyclooxygenase-2 Promotes Tumor Lymphangiogenesis and Lymph Node Metastasis in Oral Squamous Cell Carcinoma. Int. J. Oncol..

[185] Zhang J.-R., Zhu R.-H., Han X.-P. (2018). MiR-140-5p Inhibits Larynx Carcinoma Invasion and Angiogenesis by Targeting VEGF-A. Eur. Rev. Med. Pharmacol. Sci..

[186] Zhang T., Liu M., Wang C., Lin C., Sun Y., Jin D. (2011). Down-Regulation of MiR-206 Promotes Proliferation and Invasion of Laryngeal Cancer by Regulating VEGF Expression. Anticancer Res..

[187] Ribatti D. (2017). Epithelial-Mesenchymal Transition in Morphogenesis, Cancer Progression and Angiogenesis. Exp. Cell Res..

[188] Mojtahedi Z., Khademi B., Hashemi S.B., Abtahi S.M.B., Ghasemi M.A., Fattahi M.J., Ghaderi A. (2011). Serum Interleukine-6 Concentration, but Not Interleukine-18, Is Associated with Head and Neck Squamous Cell Carcinoma Progression. Pathol. Oncol. Res. POR.

[189] Duffy S.A., Taylor J.M.G., Terrell J.E., Islam M., Li Y., Fowler K.E., Wolf G.T., Teknos T.N. (2008). Interleukin-6 Predicts Recurrence and Survival among Head and Neck Cancer Patients. Cancer.

[190] Yadav A., Kumar B., Datta J., Teknos T.N., Kumar P. (2011). IL-6 Promotes Head and Neck Tu-mor Metastasis by Inducing Epithelial-Mesenchymal Transition via the JAK-STAT3-SNAIL Signaling Pathway. Mol. Cancer Res..

[191] Hao W., Zhu Y., Zhou H. (2013). Prognostic Value of Interleukin-6 and Interleukin-8 in Larynge-al Squamous Cell Cancer. Med. Oncol..

[192] Nikakhlagh S., Ranjbari N., Khorami E., Saki N. (2015). Association between Serum Levels of Interleukin-6 and Stage of Laryngeal Cancer. Iran. J. Otorhinolaryngol..

[193] Weng Y.-S., Tseng H.-Y., Chen Y.-A., Shen P.-C., Al Haq A.T., Chen L.-M., Tung Y.-C., Hsu H.-L. (2019). MCT-1/MiR-34a/IL-6/IL-6R Signaling Axis Promotes EMT Progression, Cancer Stemness and M2 Macrophage Polarization in Triple-Negative Breast Cancer. Mol. Cancer.

[194] Rokavec M., Öner M.G., Li H., Jackstadt R., Jiang L., Lodygin D., Kaller M., Horst D., Ziegler P.K., Schwitalla S. (2014). IL-6R/STAT3/MiR-34a Feedback Loop Promotes EMT-Mediated Colorectal Cancer Invasion and Metastasis. J. Clin. Investig..

[195] Yang Y., Wang W., Chang H., Han Z., Yu X., Zhang T. (2019). Reciprocal Regulation of MiR-206 and IL-6/STAT3 Pathway Mediates IL6-Induced Gefitinib Resistance in EGFR-Mutant Lung Cancer Cells. J. Cell. Mol. Med..

[196] Li F., Gu C., Tian F., Jia Z., Meng Z., Ding Y., Yang J. (2016). MiR-218 Impedes IL-6-Induced Prostate Cancer Cell Proliferation and Invasion via Suppression of LGR4 Expression. Oncol. Rep..

[197] Misso G., Zarone M.R., Lombardi A., Grimaldi A., Cossu A.M., Ferri C., Russo M., Vuoso D.C., Luce A., Kawasaki H. (2019). MiR-125b Upregulates MiR-34a and Sequentially Activates Stress Adaption and Cell Death Mechanisms in Multiple Myeloma. Mol. Ther. Nucleic Acids.

[198] Zhao X., Zhang W., Liang H., Ji W. (2013). Overexpression of MiR-155 Promotes Proliferation and Invasion of Human Laryngeal Squamous Cell Carcinoma via Targeting SOCS1 and STAT3. PLoS ONE.

[199] Zhao X., Zhang W., Ji W. (2018). MiR-196b Is a Prognostic Factor of Human Laryngeal Squamous Cell Carcinoma and Promotes Tumor Progression by Targeting SOCS2. Biochem. Biophys. Res. Commun..

[200] Wang B., Lv K., Chen W., Zhao J., Luo J., Wu J., Li Z., Qin H., Wong T.-S., Yang W. (2016). MiR-375 and MiR-205 Regulate the Invasion and Migration of Laryngeal Squamous Cell Carcinoma Synergistically via AKT-Mediated EMT. BioMed Res. Int..

[201] Fiorella R., Assennato G., Di Nicola V., Troia M., Colucci G.A., Resta L. (1991). Multivariate Analysis of Metastasis Risk in Laryngeal Carcinoma. II. Immune Response. Boll. Della Soc. Ital. Biol. Sper..

[202] Stockmann C., Schadendorf D., Klose R., Helfrich I. (2014). The Impact of the Immune System on Tumor: Angiogenesis and Vascular Remodeling. Front. Oncol..

[203] Davoine F., Lacy P. (2014). Eosinophil Cytokines, Chemokines, and Growth Factors: Emerging Roles in Immunity. Front. Immunol..

[204] Gonzalez H., Hagerling C., Werb Z. (2018). Roles of the Immune System in Cancer: From Tumor Initiation to Metastatic Progression. Genes Dev..

[205] Feng Y., Zhang B., Tan H., Su H., He W. (2001). Relationship among lymphatic metastasis, pericancerous lymphocytic reaction and dendritic cell infiltration in laryngeal carcinoma cells. Zhonghua Er Bi Yan Hou Ke Za Zhi.

[206] Remedi M.M., Donadio A.C., Chiabrando G.A. (2009). Polymorphonuclear Cells Stimulate the Migration and Metastatic Potential of Rat Sarcoma Cells. Int. J. Exp. Pathol..

[207] Fridlender Z.G., Sun J., Kim S., Kapoor V., Cheng G., Ling L., Worthen G.S., Albelda S.M. (2009). Polarization of Tumor-Associated Neutrophil Phenotype by TGF-Beta: “N1” versus “N2” TAN. Cancer Cell.

[208] Burkholder B., Huang R.-Y., Burgess R., Luo S., Jones V.S., Zhang W., Lv Z.-Q., Gao C.-Y., Wang B.-L., Zhang Y.-M. (2014). Tumor-Induced Perturbations of Cytokines and Immune Cell Networks. Biochim. Biophys. Acta.

[209] Wang R., Guo Y., Ma H., Feng L., Wang Q., Chen X., Lian M., Wang H., Fang J. (2016). Tumor Necrosis Factor Superfamily Member 13 Is a Novel Biomarker for Diagnosis and Prognosis and Promotes Cancer Cell Proliferation in Laryngeal Squamous Cell Carcinoma. Tumour Biol. J. Int. Soc. Oncodevelopmental Biol. Med..

[210] Chen L., Guan H., Gu C., Cao Y., Shao J., Wang F. (2016). MiR-383 Inhibits Hepatocellular Carcinoma Cell Proliferation via Targeting APRIL. Tumour Biol. J. Int. Soc. Oncodevelopmental Biol. Med..

[211] Kara M., Uysal S., Altinişik U., Cevizci S., Güçlü O., Dereköy F.S. (2017). The Pre-Treatment Neutrophil-to-Lymphocyte Ratio, Platelet-to-Lymphocyte Ratio, and Red Cell Distribution Width Predict Prognosis in Patients with Laryngeal Carcinoma. Eur. Arch. OtoRhinoLaryngol..

[212] Eskiizmir G., Uz U., Onur E., Ozyurt B., Karaca Cikrikci G., Sahin N., Oran A., Celik O. (2019). The Evaluation of Pretreatment Neutrophil-Lymphocyte Ratio and Derived Neutrophil-Lymphocyte Ratio in Patients with Laryngeal Neoplasms. Braz. J. Otorhinolaryngol..

[213] Marchi F., Missale F., Incandela F., Filauro M., Mazzola F., Mora F., Paderno A., Parrinello G., Piazza C., Peretti G. (2019). Prognostic Significance of Peripheral T-Cell Subsets in Laryngeal Squamous Cell Carcinoma. Laryngoscope Investig. Otolaryngol..

[214] Drennan S., Stafford N.D., Greenman J., Green V.L. (2013). Increased Frequency and Suppressive Activity of CD127low/− Regulatory T Cells in the Peripheral Circulation of Patients with Head and Neck Squamous Cell Carcinoma Are Associated with Advanced Stage and Nodal Involvement. Immunology.

[215] Chen B., Zhang D., Zhou J., Li Q., Zhou L., Li S.-M., Zhu L., Chou K.-Y., Zhou L., Tao L. (2013). High CCR6/CCR7 Expression and Foxp3+ Treg Cell Number Are Positively Related to the Progression of Laryngeal Squamous Cell Carcinoma. Oncol. Rep..

[216] Lu E., Su J., Zhou Y., Zhang C., Wang Y. (2017). CCL20/CCR6 Promotes Cell Proliferation and Metastasis in Laryngeal Cancer by Activating P38 Pathway. Biomed. Pharmacother. Biomedecine Pharmacother..

